# Microglia Regulate Neuronal Circuits in Homeostatic and High-Fat Diet-Induced Inflammatory Conditions

**DOI:** 10.3389/fncel.2021.722028

**Published:** 2021-10-13

**Authors:** Xiao-Lan Wang, Lianjian Li

**Affiliations:** ^1^Department of Nephrology, Union Hospital, Tongji Medical College, Huazhong University of Science and Technology, Wuhan, China; ^2^Department of Surgery, Hubei Provincial Hospital of Traditional Chinese Medicine, Wuhan, China; ^3^Hubei Province Academy of Traditional Chinese Medicine, Wuhan, China

**Keywords:** microglia, cognition, obesity, neuronal circuits, phagocytosis, inflammation

## Abstract

Microglia are brain resident macrophages, which actively survey the surrounding microenvironment and promote tissue homeostasis under physiological conditions. During this process, microglia participate in synaptic remodeling, neurogenesis, elimination of unwanted neurons and cellular debris. The complex interplay between microglia and neurons drives the formation of functional neuronal connections and maintains an optimal neural network. However, activation of microglia induced by chronic inflammation increases synaptic phagocytosis and leads to neuronal impairment or death. Microglial dysfunction is implicated in almost all brain diseases and leads to long-lasting functional deficiency, such as hippocampus-related cognitive decline and hypothalamus-associated energy imbalance (i.e., obesity). High-fat diet (HFD) consumption triggers mediobasal hypothalamic microglial activation and inflammation. Moreover, HFD-induced inflammation results in cognitive deficits by triggering hippocampal microglial activation. Here, we have summarized the current knowledge of microglial characteristics and biological functions and also reviewed the molecular mechanism of microglia in shaping neural circuitries mainly related to cognition and energy balance in homeostatic and diet-induced inflammatory conditions.

## Introduction

Microglia are derived from primitive myeloid progenitors in the early fetal development stage and distribute throughout the central nervous system (CNS) parenchyma (Ginhoux et al., 2010). Since the formation of the blood-brain barrier (BBB), microglia are isolated from the periphery under normal conditions (Mildner et al., 2007). They are longevity and self-renewal cells, with a low turnover rate under homeostatic conditions across the lifespan (Fuger et al., 2017). The development, proliferation, maintenance, and survival of microglia mainly depend on colony-stimulating factor 1 (CSF1), which is secreted by neurons, as well as glial cells (Elmore et al., 2014). Microglia constitute approximately 10% of all cells in the rodent brain and their density shows a wide variation between different brain regions (Tan et al., 2020). The volume of the cell body and the length and complexity of the processes in microglia are changeable and closely related to their function (Streit et al., 1999; Ayoub and Salm, 2003; Fernandez-Arjona et al., 2017; Ayata et al., 2018). Microglia are highly motile cells and the brain parenchyma can be completely screened by resting microglia every few hours in the homeostatic mouse brain (Nimmerjahn et al., 2005).

As the brain’s resident macrophages, microglia serve as the scavengers to maintain the brain’s homeostasis, such as the engulfment of cellular debris and dead cells (Colonna and Butovsky, 2017). Microglia continuously extend and retract their highly ramified processes to survey the surrounding synapses, and play a crucial role in shaping and maintaining the optimal synaptic network (Wake et al., 2009; Paolicelli et al., 2011; Ji et al., 2013; Wu et al., 2015; Gao et al., 2018). The neural circuit is highly dynamic, including new synaptic formation, synaptic strengthening, and selective synaptic elimination (Hua and Smith, 2004; Caroni et al., 2014). The pruning of the inappropriate or weak synapses is a necessary process for appropriate brain connectivity, which strengthens more productive connections and efficient neural networks (Caroni et al., 2014; Jiang and Nardelli, 2016). Microglia phagocytosis plays an important role in the process of synaptic maturation (Paolicelli et al., 2011; Schafer et al., 2012). Additionally, microglia are involved in synaptic formation and neurogenesis in the brain (Miyamoto et al., 2016; Weinhard et al., 2018). According to these functions, microglia play a critical role in learning and memory processes and control of energy homeostasis (Paolicelli et al., 2011; Garcia-Caceres et al., 2019).

Furthermore, microglia are innate immune cells of the brain, which defend against infectious agents, neuronal damage, and the pathogenic protein aggregates produced in neurodegenerative diseases by phagocytosing and initiating immune responses (Gonzalez-Scarano and Baltuch, 1999; Aloisi, 2001; Davalos et al., 2005; Subhramanyam et al., 2019). To deal with these situations, microglia produce pro-inflammatory cytokines (TNFα, IL-1β, and IL-6), chemokine ligand 2 (CCL2), superoxide, reactive oxygen species (ROS), nitric oxide (NO), and matrix metallopeptidase 12 (MMP12), which subsequently induce inflammation, neuronal loss, and brain damage. Microglia also produce anti-inflammatory cytokines (IL-10 and IL-4) to facilitate phagocytosis of cell debris and promote the reconstruction of the extracellular matrix (ECM) and tissue repair (Sarlus and Heneka, 2017; Subhramanyam et al., 2019). Additionally, microglia secrete neurotrophic factors, such as brain-derived neurotrophic factor (BDNF), transforming growth factor-beta 1 (TGF-β), and insulin-like growth factor 1 (IGF-1) to support neuronal health and survival (Coull et al., 2005; Olah et al., 2011; Walker et al., 2014; Orihuela et al., 2016).

Besides, microglia express a variety of inducible receptors that regulate immune response and phagocytosis of microglia, promote migration, and modulate neural networks (Aloisi, 2001; Hickman et al., 2018; Das and Chinnathambi, 2019; Subhramanyam et al., 2019). These receptors include complement receptors (CRs) (CR1 and CR3) (Heneka et al., 2015), chemokine receptors (C-X3-C motif chemokine receptor 1 (Cx3cr1) and CXCR4) (El Khoury et al., 2007), cytokine receptors, CSF1 receptor (CSF1R), Toll-like receptors (TLRs) (TLR4 and TLR1/2), NOD-like receptor (NLR) (NLRP3 inflammasome) (Heneka et al., 2015), scavenger receptors (CD36, SR1, and MARCO) (Areschoug and Gordon, 2009), and TAM receptors (tyrosine kinases Mer and AxL) (Lemke, 2013).

Recently, accumulating studies based on the novel technologies revealed the intrinsic heterogeneity of microglia, including the spatial and temporal heterogeneity, the distinctive features in the same brain region, as well as different responses to the identical stimuli (Sousa et al., 2018; Masuda et al., 2019; Stratoulias et al., 2019; Tan et al., 2020; Mendes et al., 2021; Young et al., 2021). For example, in homeostatic conditions, cerebellar microglia display a phagocytic phenotype while striatum microglia show a surveillance state, which depends on the rate of neuronal apoptosis in the adult mice brain (Ayata et al., 2018). *CD11c* positive microglia represent during the postnatal day (P)3-P5, and then drop to few population in adult mice, which may play a crucial role in myelination and neurogenesis during development (Wlodarczyk et al., 2017). Ultrastructural analyze using electron microscopy (EM) uncovered the “dark” microglia, which display signs of oxidative stress and are distinct from the surrounding normal microglia (Bisht et al., 2016); dark microglia are more active in engulfing synaptic elements than normal populations and involve in the neuronal circuit remodeling, which even increases under chronic stress and aging conditions (Bisht et al., 2016; Mendes et al., 2021). Besides, single-cell transcriptome profiling has revealed remarkable heterogeneity of microglial cells during development, homeostatic and disease conditions in both mice and humans (Sousa et al., 2018; Hammond et al., 2019; Masuda et al., 2020). Microglial subtypes, such as proliferative-region-associated microglia (PAM), axonal tract-associated microglia (ATM), triggering receptor expressed on myeloid cells 2 (TREM2)-microglia, TREM2-independent microglia, and neurodegenerative disease-associated microglia (DAM) have been discovered recently (Wang et al., 2015; Keren-Shaul et al., 2017; Sousa et al., 2018; Li et al., 2019; Stratoulias et al., 2019).

Microglial activation damages the surrounding healthy neural tissue; while the dead or dying neurons secreted factors (such as ATP) in turn exacerbate the activation of microglia. These processes cause persistent chronic neuroinflammation and neuronal loss, which have been observed in neurodegenerative and metabolic diseases such as Alzheimer’s disease, Parkinson’s disease, Huntington’s disease, amyotrophic lateral sclerosis, and obesity (Hide et al., 2000; Fu et al., 2014; Tang and Le, 2016; Hickman et al., 2018; Rajendran and Paolicelli, 2018). Aging microglia display an increased phagocytosis, which may also contribute to the development of neurodegenerative diseases (Sierra et al., 2007; Hickman et al., 2013). Moreover, HFD consumption triggers microglial activation and inflammation, which leads to energy imbalance (i.e., obesity) and cognitive decline in both adult humans and rodents (Murray et al., 2009; Pistell et al., 2010; Edwards et al., 2011; Holloway et al., 2011; Heyward et al., 2012; Thaler et al., 2012). Obesity is a serious global health problem and has been regarded as a clinical risk factor for cognitive disorders later in life (Yau et al., 2012; Morris et al., 2015; Di Cesare et al., 2016; Pedditizi et al., 2016). Increasing literature indicated that a long-term HFD consumption affects the overall physiology, including insulin resistance, hippocampal oxidative stress, chronic systemic inflammation, neuroinflammation, microglial activation, and hippocampal oxidative stress; the complex interplays among these pathogenic factors contribute to cognitive impairment (Bruce-Keller et al., 2010; Spencer et al., 2017; Guo et al., 2020). Neuroimaging studies showed that obese individuals have decreased brain volume, especially in brain regions linked to cognitive function (Pannacciulli et al., 2006; Raji et al., 2010). Obesity is the onset of neurodegeneration and also exacerbates the cognitive decline in aging (Valcarcel-Ares et al., 2019). Microglial activation plays a crucial role in neurodegenerative diseases and cognitive decline caused by HFD consumption (Hong et al., 2016; Jeong et al., 2019). Here, we review the microglial biological functions in regulation of neural circuits related to cognition and energy homeostasis in physiological and diet-induced inflammatory conditions.

## Microglial Function in Homeostasis

### Synapse and Cell Body Elimination

**FIGURE 1:**
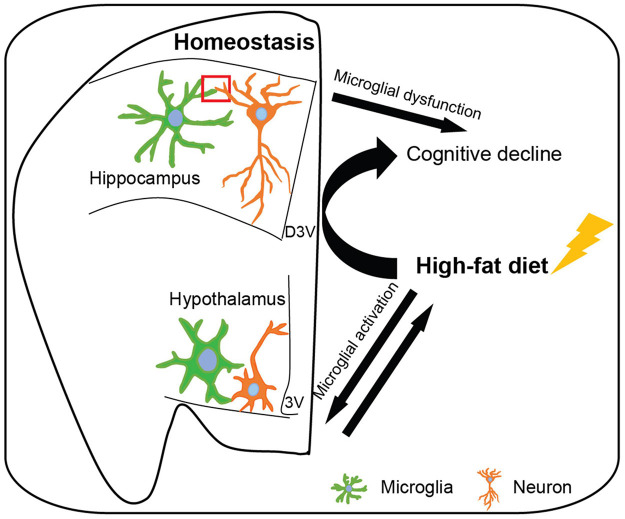
Microglia affect hippocampal and hypothalamic neuronal circuits in homeostatic and high-fat diet-induced obese conditions. In the homeostatic brain, microglia survey the surrounding microenvironment and interact with neurons to shape neuronal circuits, including synaptic phagocytosis, engulfment of unwanted cells and debris, synaptic formation, neurogenesis, secretion of soluble factor, and expression of inducible receptors. Hippocampal microglia participate in synaptic remodeling during the cognitive process and hypothalamic microglia regulate energy homeostasis, however, microglial dysfunction leads to cognitive decline and obesity, respectively. Moreover, high-fat diet (induced obesity) results in hypothalamic microglial activation which may exacerbate the progress of obesity. Additionally, high-fat diet-induced obesity activates hippocampal microglia and leads to cognitive decline. D3V, dorsal third ventricle; 3V, third ventricle.

During brain development, an excess of synapses are formed, however, many of them will be eliminated during synaptic pruning to establish mature brain connectivity (Tremblay et al., 2011; Schafer and Stevens, 2013). In the human frontal cortex, the highest synaptic density is during early postnatal development and significantly reduces with aging (Huttenlocher, 1979; Kabaso et al., 2009). In mice hippocampus, genes related to synaptic formation express from the first postnatal week and show a gradual increase until the late postnatal weeks; the peak of synaptic density is around the fourth postnatal week in mice (Mody et al., 2001; Paolicelli et al., 2011). Following the synaptic formation, the hippocampal synapses and circuits exhibit increased plasticity and maturation; this process is the important neurochemical foundation of learning and memory and crosses the whole life (Mody et al., 2001; Caroni et al., 2014). Skill learning accelerates the synaptic rearrangement process (Caroni et al., 2014). Emerging work implicates that microglia and related immune molecules are key mediators in the refinement of synaptic circuits of normal neurophysiology, particularly during early brain development (Figure 1; Schafer et al., 2013; Sierra et al., 2013; Ikegami et al., 2019; Mallya et al., 2019).

The postsynaptic density protein 95-(PSD95, a postsynaptic marker) and the synaptosomal associated protein 25 (SNAP25, a presynaptic marker) have been observed in microglia in the hippocampus at P15 mice (Paolicelli et al., 2011). While the presynaptic terminals of retinal ganglion cell (RGC) were also localized with microglia in the P5 dorsal lateral geniculate nucleus (dLGN) (Schafer et al., 2012). *In vivo* imaging revealed that microglia contact with synaptic elements in the visual cortex of juvenile mice (Tremblay et al., 2010). Additionally, a recent time-lapse fluorescence imaging study provides direct evidence that microglia engulf synaptic material in living brain tissue and this result is further confirmed in their fixed mouse hippocampal tissue at P15, the peak expression of phagosome (Weinhard et al., 2018). These findings suggest the involvement of microglia in the modification or engulfment of synaptic structures in the brain.

Microglial receptor Cx3cr1 interacts with neuronal chemokine C-X3-C motif chemokine ligand 1 (Cx3cl1) to regulate neuronal network and functions (Ransohoff and Stevens, 2011). Cx3cr1 knockout mice show a transient reduction of microglial density during postnatal day 8 (P8) to P28, which leads to an increased spine density and abundant excitatory synapses in the hippocampus during the second and third postnatal weeks (Paolicelli et al., 2011); these transgenic mice exhibit deficits in social interaction and increased repetitive behavior that has been associated with autism and other neurodevelopmental and neuropsychiatric disorders (Paolicelli et al., 2011; Zhan et al., 2014). Microglia participate in the synaptic pruning also via recognizing the classical complement cascade proteins (C1q and C3), which localize to the unwanted synapses (Stephan et al., 2012; Zabel and Kirsch, 2013; Wang et al., 2020). Genetic strategies (knockout of CR3 or C3) or pharmacological (minocycline) perturbation of microglial phagocytosis results in significantly increased synaptic density in retinal ganglion cells and deficits in eye-specific segregation in early postnatal mice (Schafer et al., 2012). Complement-dependent synapse elimination of microglia mediates the forgetting of remote memories, which can be prevented by microglial depletion or phagocytic inhibition (Wang et al., 2020). Altering microglial activation by disrupting purinergic receptor-P2Y12 abrogates ocular dominance plasticity in the binocular visual cortex, experience-dependent synaptic plasticity of the adolescent brain (Haynes et al., 2006; Sipe et al., 2016). Besides, microglial TREM2 deficient mice show impaired microglial activation and synapse elimination during early brain development and corresponding sociability defects in adulthood which is similar to autism in humans (Filipello et al., 2018). These studies indicate that microglial synaptic elimination is crucial for the formation of mature and functional neuronal circuitry during CNS development.

As we have described that deficit in microglial phagocytic function results in immature brain connection, however, increased microglial engulfment of synapses leads to spine number reduction that could also impair neural circuits. For example, vacuolar sorting protein 35 (VPS35), a component of selective endosome-to-Golgi retrieval of membrane proteins, regulates microglia immune response and phagocytosis (Lucin et al., 2013; Yin et al., 2016). Microglia specific knockout of VPS35 results in increased microglial density and activity in the hippocampus, accompanied by impaired dendritic morphology and reduced dendritic spine density in newborn neurons of the subgranular zone; microglial-neuronal co-culture revealed that VPS35-depleted microglia show increased phagocytic activity toward postsynaptic protein-PSD95, that may contribute to the loss of dendritic spines and depression-like behavior and impaired long-term recognition memory in mutant mice (Appel et al., 2018). Moreover, Polycomb repressive complex 2 (PRC2) catalyzes histone H3 lysine 27 tri-methylation (H3K27me3) and represses transcription, which epigenetically regulates microglial activation (Margueron and Reinberg, 2011; Cheray and Joseph, 2018). Microglia-specific loss of PRC2 increases the clearance activity, which leads to a reduction of spine density in the striatal medium spiny neuron, accompanied by enhanced anxiety behavior and impairments of cognition and memory in adult mice (Ayata et al., 2018). However, microglia-specific Brain and Muscle Arnt-like 1 (Bmal1) deficiency enhances microglial phagocytosis, which may be associated with the formation of mature spines in the hippocampus in mice during the learning process and also relate to less pro-opiomelanocortin (POMC)-neuronal loss in the mediobasal hypothalamus in mice on a HFD (Wang et al., 2021). *In vitro* experiment shows that microglia-like cells derived from schizophrenia patients exhibit excessive synaptic pruning, which may underlie the reduced synapse density found in postmortem cortical tissue of schizophrenia patients (Sellgren et al., 2019). The antibiotic minocycline which can reduce microglial activation and engulfment of synapse structures may have therapeutic benefits in schizophrenia patients (Tikka et al., 2001; Sellgren et al., 2019). Therefore, appropriate microglial phagocytic capacity is crucial for synaptic pruning and mature patterns of neuronal connectivity.

In addition, microglia engulf apoptotic neurons to maintain CNS hemostasis, which has been seen in both postnatal and adult mouse brain regions (Ashwell, 1990; Ayata et al., 2018). For example, in the subgranular zone, where neurogenesis occurs, most of the newborn cells are eliminated through apoptosis-coupled phagocytosis by microglia; only a small amount of neuroblasts survive and integrate into neuronal circuits (Sierra et al., 2010). Microglia also engulf live neural progenitor cells (NPCs) and remove viable neurons to limit the overproduction of neurons during brain development (Cunningham et al., 2013; Brown and Neher, 2014; Sellgren et al., 2017). Except these, microglial phagocytose ALDH1L1, which is an early astrocyte marker, indicating the engulfment of astrocytic material in the early developing amygdala, which subsequently affects the neural circuitry and mice behavior (VanRyzin et al., 2019). These findings suggest that microglia not only clean away apoptotic cells to maintain a healthy microenvironment for neuronal survival but also regulate the live cells to ensure the proper cell number and brain circuits.

### Spine Formation

Increasing evidence indicated that microglia are involved in synaptic formation and neurogenesis, which are important in brain circuits’ maturation (Reshef et al., 2017). Post-mortem human studies showed that at early gestational weeks, microglia prominently accumulate at the cortical plate-subplate junction, where the first synapses were found, suggesting that microglia may be involved in synaptic formation (Verney et al., 2010). A study showed that microglial contact promotes the new protrusion formation from the head of the spine and increases the spine length, which is direct evidence that microglia are involved in structural synaptic plasticity in developing mice brain (Weinhard et al., 2018). This phenomenon is also observed in pyramidal neurons in the developing somatosensory cortex. The *in vivo* imaging showed that microglia induce spine formation by creating contact with dendrites in a period of intense synaptogenesis (P8–10) in layer II/III pyramidal neurons; transient genetic ablation of microglia around P8 reduces the spine density and the frequency of miniature excitatory postsynaptic currents (mEPSCs) in these neurons, demonstrating that microglia promote functional mature synaptic formation and subsequently contribute to the maturation of specific neuronal circuits (Miyamoto et al., 2016). Additionally, transient microglial depletion in the late postnatal period (P19) or young adulthood (P30) causes a significant decrease in both spine formation and elimination in layer V pyramidal neurons in the motor cortex, which leads to deficits in multiple learning tasks and learning-induced synaptic remodeling in mice (Parkhurst et al., 2013). These findings suggest that microglia directly regulate synaptic formation and maturation.

Besides, microglia impact synaptic formation and plasticity by releasing soluble factors, such as inflammatory cytokines, ROS, NO, and neurotrophic factors (Vezzani and Viviani, 2015). Hippocampal neurons and microglial co-cultures show that microglia induce neuronal synaptic formation via releasing interleukin 10 (IL-10) which can interact with IL-10 receptors expressed on the hippocampal neurons (Lim et al., 2013). Microglia mediate synaptic transmission and strength via CR3-NADPH oxidase cascade (Collingridge and Peineau, 2014; Zhang et al., 2014). It has been suggested that a physiological level of IL-1β is necessary for the induction and maintenance of long-term potentiation (LTP), but excessive IL-1β has an inhibitory effect on synaptic strength (Bellinger et al., 1993; Yirmiya et al., 2002; Avital et al., 2003). Increase IL-1β secretion in the hippocampus lead to the impairment of synaptic plasticity, cognitive deficits, and mood disorders, which has been seen in Cx3cr1 deficient adult mice (Rogers et al., 2011). Cx3cr1 deficiency also delays the functional maturation of postsynaptic glutamate receptors of the thalamocortical synapses in the barrel cortex in mice (Hoshiko et al., 2012). Microglia-specific BDNF depleted mice repeat many phenotypes generated by microglia depletion, indicating that microglia BDNF is an important factor for motor learning and memory-associated synaptic remodeling (Parkhurst et al., 2013). Moreover, microglial deficiency of KARAP/DAP12, a transmembrane polypeptide associated with cell-surface receptors, leads to the decreased expression of glutamate receptor content in the postsynaptic and expression of synaptic tyrosine kinase receptor B (Roumier et al., 2004). These observations indicate that the interaction between microglia and neurons controls the functional maturation of synapses.

### Neurogenesis

Newborn neurons are added to neural circuits throughout life, which is an important part to maintain brain homeostasis. Postnatal neurogenesis mainly occurs in the subventricular zone (SVZ) of the lateral ventricles and subgranular zone (SGZ) of the hippocampus and plays a critical role in learning and memory (Ernst et al., 2014). During this process, microglia are essential for maintaining the proper neuronal cell number in the neural network (de Miranda et al., 2017). For example, activated microglia release cytokines to enhance neurogenesis and oligodendrogenesis of SVZ in P1–P10 rat brain; *in vitro* assays showed that neurogenesis can be enhanced by activating microglia or independently adding the cytokines (IL-4, low concentration of IL-1β, IFN-γ, or IFN- γ) in the culture medium of (NPCs) (Butovsky et al., 2006; Shigemoto-Mogami et al., 2014). Microglia-conditioned medium also directs the migration of precursor cells and influences the differentiation of both adult and embryonic neural precursor cells toward a neuronal phenotype (Aarum et al., 2003). In addition, this conditioned medium enhances cerebellar granule cell survival and promotes the proliferation of precursor neurons, which is mediated by the mitogen-activated protein kinase (MAPK) and phosphatidylinositol-3-kinase (PI3K)/Akt signal transduction pathways (Morgan et al., 2004). Microglia produce IGF1 to support cortical neuronal survival (Ueno et al., 2013). Furthermore, microglia-neuronal Cx3cr1-Cx3cl1 signaling mediates the exercise-induced activation of NPCs in the hippocampus and the suppression of NPCs in the aged brain (Vukovic et al., 2012); microglial Cx3cr1 also enhances neurogenesis in the dentate gyrus (DG) via inhibiting sirtuin 1 (SIRT1)/NF-kB p65 signaling (Sellner et al., 2016). Cx3cr1 deficiency impairs the maturation of newborn granule neurons in the adult brain (Bolos et al., 2018). Replacement of microglia in aged mice by manipulating CSF1R restores microglial cell densities and morphologies to those found in young adult animals, without alteration of immune reactivity; the microglial repopulation improves the synaptogenesis related gene expression, reverses the hippocampal neuronal complexity, and fully rescues the age-induced deficits in LTP and spatial memory in mice (Elmore et al., 2018).

Besides, neurogenesis has been also observed in postnatal and adult mammalian hypothalamus and is involved in the control of energy homeostasis (Pierce and Xu, 2010; Yoo and Blackshaw, 2018). A study indicated that the fibroblast growth factor 10-expressing (Fgf10 +) tanycytes are the hypothalamic progenitor cells and can generate neurons and astrocytes to populate the ARC in mice (Haan et al., 2013). Infusion of BDNF into the lateral ventricle stimulates hypothalamic neurogenesis (Pencea et al., 2001). GPR40 (FFAR1), the receptor for medium and long chain unsaturated fatty acids, also modulates hypothalamic cell proliferation and survival through p38 and BDNF in adult mice (Engel et al., 2020). Additionally, administration of ciliary neurotrophic factor (CNTF) into the lateral ventricle induces hypothalamic neuronal and glial proliferation and reduces body weight in HFD fed mice (Kokoeva et al., 2005). CNTF is a tropic factor for neurons but also stimulates microglia to secrete glial cell line-derived neurotrophic factor (GDNF) and indirectly promote neuronal survival (Krady et al., 2008; Lin et al., 2009). Moreover, microglia release BDNF to support the surrounding neurons that regulate appetite; mice with BDNF deficiency in microglia show hyperphagia and obesity (Urabe et al., 2013). Besides, transient removal of microglia from the embryonic brain leads to decreased litter size and increased mortality of pups at early postnatal days; while survived pups show significant accumulation of apoptotic POMC neurons in the hypothalamus, and accelerated body weight gain since P5 (Rosin et al., 2018). These studies indicate that microglia are closely associated with neurogenesis and the regulation of neuronal circuits.

## Microglia Regulate Energy Metabolism

Energy homeostasis is the biological process that maintains the energy balance by regulating food intake and energy expenditure (Bray et al., 2012). A growing number of findings have demonstrated the important role of CNS in the control of energy homeostasis, especially the neuronal circuits in the hypothalamic arcuate nucleus (ARC) (Myers and Olson, 2012; Abdalla, 2017; Garcia-Caceres et al., 2019). The ARC is located in the mediobasal hypothalamus (MBH), where close the third ventricle and median eminence (ME). The ME is one of the circumventricular organs (CVOs) that are comprised of extensive and highly permeable capillaries to facilitate the diffusion of specific molecules found in the blood; this characteristic allows the peripheral metabolic signals to be sensed by adjacent neuronal dendrites that extend from the ARC (Djogo et al., 2016). Two important populations of neurons in the ARC are involved in this process, the orexigenic agouti-related peptide (AgRP)/neuropeptide Y (NPY) neurons and the POMC/cocaine and amphetamine regulated transcript (CART) neurons (Schwartz et al., 2000; Palkovits, 2003; Luquet et al., 2005; Morton et al., 2006; Krashes et al., 2011). Activation of the AgRP/NPY neurons stimulates food intake and reduces energy expenditure, thereby increasing body weight, whereas activation of POMC neurons has the opposite effect on food intake, energy expenditure, and body weight (Hahn et al., 1998; Ellacott and Cone, 2004; Lechan and Fekete, 2006; Toda et al., 2017).

Cumulative studies indicate that hypothalamic microglia are implicated in the maintenance of neural circuits and regulate energy homeostasis. Active microglia are more adjacent to POMC neurons than to NPY neurons in the hypothalamus, thus may have a more powerful influence on POMC cells (Gao et al., 2017). The POMC neurons release alpha-melanocyte-stimulating hormone (α-MSH) and cocaine-and amphetamine-regulated transcript (CART) peptides to promote energy expenditure and satiety; α-MSH derives from POMC-a precursor peptide and binds to melanocortin receptors-MC3R and MC4R-in the paraventricular nucleus (PVN) and the nucleus of the solitary tract (NST) (Wang et al., 2000; Pinto et al., 2004; Mountjoy, 2010; Lau and Herzog, 2014; Koch et al., 2015). Microglial leptin receptor-deficiency impairs microglial phagocytic capacity, which contributes to the decreased POMC neurons, reduced α-MSH projection from the ARC to PVN, and increased food intake and body weight gain in mice (Gao et al., 2018). Moreover, microglia release neurotrophic factors to support the surrounding neurons that regulate appetite; mice with BDNF deficiency in microglia show hyperphagia and obesity (Urabe et al., 2013).

High-fat diet (HFD)-induced mediobasal hypothalamic microglial activation and inflammation has been linked to the impairment of brain circuits in control of energy balance, as well as the development and progression of obesity and (pre)diabetes in both humans and rodents (Gao et al., 2014; Baufeld et al., 2016; Jais and Bruning, 2017; Le Thuc et al., 2017; Avalos et al., 2018). Specifically, HFD consumption induces low-grade hypothalamic inflammation, neuronal stress, and leptin resistance, which is accompanied by rapid glial cell accumulation, especially microglial cells (De Souza et al., 2005; Thaler et al., 2012; Valdearcos et al., 2014). Hypothalamic inflammatory gene expression was significantly increased during 1–3 days of HFD feeding, while microglial activation in the ARC starts as early as 3 days after HFD exposure, prior to body weight gain in both rats and mice (Thaler et al., 2012; Baufeld et al., 2016; Kim et al., 2019). The time course of HFD feeding induced-hypothalamic informatory gene expression as well as microglial activation and proliferation has been presented in Table 1. HFD leads to neuronal injury in the ARC since the first week of feeding (Thaler et al., 2012; Kim et al., 2019). Eight weeks of HFD consumption induces neuronal apoptosis and a loss of synaptic inputs in the ARC and lateral hypothalamus in rodents (Moraes et al., 2009; Horvath et al., 2010; Kim et al., 2019). Inhibition of microglial activation and inflammation in the ARC may be sufficient to protect against diet-induced hypothalamic inflammation and POMC neuronal loss and prevent obesity (Valdearcos et al., 2014; Andre et al., 2017). It has been shown that the hypercaloric diet-induced obese mice show persistently activated microglia in the MBH with increased secretion of TNFα, which improves the mitochondrial stress of POMC neurons and contributes to the development of obesity; specific knocking down of the TNFα downstream signals in the MBH of DIO mice reduces the body weight (Yi et al., 2017). HFD feeding also alters microglial mitochondrial morphology and increases the expression of uncoupling protein 2 (UCP2)-a mitochondrial protein involved in reactive oxygen species (ROS) generation and fuel utilization, which is associated with increased hypothalamic inflammation (Kim et al., 2019). Deletion of microglial UCP2 affects mitochondrial function, ameliorates microglial activation in the ARC, alters the POMC synaptic input organization and neuronal activation, and leads to less body weight gain under HFD conditions (Kim et al., 2019).

**TABLE 1:** Time course of HFD consumption induced-hypothalamic inflammation and microglial activation in the ARC.

	**Time course**	**1 day**	**3 days**	**1 weeks**	**2 weeks**	**4 weeks**	**8 weeks**	**16 weeks**	**20 weeks**	**References**
Hypothalamic inflammation	In rats	↑	↑	−	↑	↑			↑	Thaler et al., 2012
	In mice	↑	↑/−	↑/−		↑/−	−			Thaler et al., 2012; Valdearcos et al., 2014; Baufeld et al., 2016; Kim et al., 2019
Microglial activation	In rats	−	↑	↑	↑	↑	↑			Thaler et al., 2012
	In mice		↑/−	↑/−		↑/−	↑	↑		Valdearcos et al., 2014; Baufeld et al., 2016; Kim et al., 2019

*↑ and − indicate an increase and no alteration after HFD exposure, respectively.*

Additionally, depletion of microglia or selective silencing of microglial NF-κB-mediated signaling in the transgenic mouse model (microglial specific IKKβ knockout) reduces microgliosis and greatly limits diet-induced hyperphagia and weight gain (Valdearcos et al., 2017). Activating microglia through cell-specific deletion of the negative NF-κB regulator A20 induces spontaneous microgliosis in MBH, reduces energy expenditure, and increases food intake and weight gain even independent of the HFD challenge (Valdearcos et al., 2017). Besides, inhibition of the microglial toll-like receptor-4 (TLR4) prevents the central orexigenic AgRP/NPY neuronal response and feeding behavior (Reis et al., 2015). HFD induced hypothalamic inflammatory response and impaired insulin-signaling pathway are mediated by activation of hypothalamic c-Jun N-terminal kinase (JNK) and nuclear factor-kappaB (NF-κB) signaling pathways (De Souza et al., 2005; Zhang et al., 2008). Specific inhibition of hypothalamic JNK restores insulin signaling and reduces caloric intake and weight gain in HFD fed rats (De Souza et al., 2005). Surprisingly, conditionally knockout lipoprotein lipase in microglia in adult mice leads to reduced immune response and phagocytosis, which results in more POMC neuronal loss and body weight gain than controls when challenged with HFD (Gao et al., 2017). Thus, proper microglial functions are necessary for maintaining the appropriate hypothalamic neuronal circuits to regulate energy metabolism.

Besides, microglia modulate the function of astrocytes in the brain (Greenhalgh et al., 2020). Astrocytes regulate synaptic transmission and neuronal activity and support neuronal survival and synaptogenesis (Araque et al., 2014). It’s known that astrocytes regulate hypothalamic neuronal circuits in control of feeding; astrocyte-specific loss of leptin receptors alters astrocyte morphology and synaptic inputs onto hypothalamic neurons and enhances fasting and ghrelin-induced food intake in mice (Kim et al., 2014). Consumption of HFD also induces astrocyte activation in the ARC, which is associated with hypothalamic inflammation and synaptic loss of POMC neurons in both humans and rodents (Horvath et al., 2010; Thaler et al., 2012; Valdearcos et al., 2014; Baufeld et al., 2016; Kim et al., 2019). A recent study showed that astrocytes directly engulf apoptotic neurons to maintain brain homeostasis (Damisah et al., 2020), which may also occur in HFD-induced inflammatory conditions. Additionally, astrocytes are involved in innate immunity. Neurotoxic reactive astrocytes can be induced by microglia secreted inflammatory cytokines and mediate complement-dependent neuronal loss (Liddelow et al., 2017; Fakhoury, 2018). HFD consumption increases the expression of *Myd88*, an adaptor molecular of TLR signaling, in hypothalamic astrocytes; astrocyte-specific deletion of *Myd88* ameliorates hypothalamic reactive gliosis and inflammation and prevents obesity in HFD fed mice (Jin et al., 2020). Astrocyte-specific knockout of IKKβ, a crucial cofactor of NF-κB-mediated inflammation, also reduces hypothalamic inflammation and astrocytosis, as well as protects mice from weight gain under HFD conditions (Douglass et al., 2017). However, a study showed that HFD feeding only induces hypothalamic microglial inflammatory activation, but not astrocytes (Valdearcos et al., 2014). Activated microglia in HFD fed mice might be upstream regulators in the synaptic plasticity of the POMC neurons and astrogliosis in the ARC (Kim et al., 2019).

Moreover, microglial activation recruits peripheral myeloid cells, such as monocytes, perivascular, and meningeal macrophages into the mediobasal hypothalamus in mice; while this phenomenon is abolished when depleting of the resident microglia by PLX5622 (Valdearcos et al., 2017). The activation and expansion of infiltrated myelod cells participates in the mediobasal hypothalamic microglosis and inflammation in HFD fed mice (Valdearcos et al., 2017; Lee et al., 2018). Additionally, HFD-induced inflammation impairs the structure and function of hypothalamic endothelial cells and tanycytes, which promotes the permeability and disrupts the integrity of blood-brain barrier (BBB) (Freeman and Granholm, 2012; Garcia-Prieto et al., 2015; Ramalho et al., 2018). BBB dysfunction increased infiltration of immune cells, such as lymphocytes and neutrophils, and HFD-induced peripheral inflammatory mediators may exacerbate the hypothalamic microglial activation and inflammation (Argaw et al., 2012; Zenaro et al., 2015; Duan et al., 2018; Edison, 2020). The reciprocal interplay between microglia and infiltrating immune cells impairs hypothalamic neurons control of feeding behavior and energy expenditure under HFD conditions.

However, overexpression of an anti-inflammatory cytokine-IL-10 in mice ameliorates HFD induced-obesity, restores *POMC* expression, and attenuates the leptin resistance by inhibiting IKKs activation and SOCS3 expression in ARC (Nakata et al., 2017). Quercetin, a polyphenolic flavonoid, is known to protect against obesity-induced oxidative stress and inflammation in peripheral tissues (Le et al., 2014; Kim et al., 2015). A study has reported that Quercetin reduces hypothalamic inflammation by inhibiting the microglia-mediated inflammatory responses in obese mice and also upregulates the expression of the antioxidant enzyme heme oxygenase (HO-1) in the hypothalamus (Yang et al., 2017). These findings indicate that inhibiting inflammation, increasing the anti-inflammation, or preventing oxidative stress in the hypothalamus may be effective therapeutic strategies for the treatment of metabolic disorders, including obesity.

## Microglial Function in High-Fat Diet Consumption-Associated Cognitive Decline

Studies showed that HFD feeding results in cognitive impairment via microglial activation (Hao et al., 2016; Maldonado-Ruiz et al., 2017; Cope et al., 2018). Obesity increases microglial processes in the prefrontal cortex, which may contribute to the diminished dendritic spine density and synaptic protein, and impairs cognition in HFD fed rats (Bocarsly et al., 2015). Reduced dendritic spine and impaired cognition have also been observed in HFD induced obese mice, accompanied by increased microglial synaptic engulfment in the hippocampus; inhibition of microglial activation or blocking microglial phagocytosis prevent the synaptic loss and cognitive deficits in these mice (Cope et al., 2018). Besides, HFD-induced obese mice exhibit increased IL1β secretion in the hippocampus, which leads to cognitive impairment (Hao et al., 2016; Guo et al., 2020). Obesity in aged mice promotes the gene expression of microglial pro-inflammatory cytokines and exacerbates neuroinflammation and cognitive decline (Masser et al., 2014; Mangold et al., 2017; Valcarcel-Ares et al., 2019). Inducible knockout of the IL1β receptor on microglia prevents microglial activation and neuroinflammation, as well as protects obese mice from cognitive impairment (Guo et al., 2020). Therefore, microglia play a direct role in HFD consumption-induced cognitive dysfunction.

Besides, microglia have a direct effect on astrocytes activation under inflammatory conditions (Kirkley et al., 2017; Greenhalgh et al., 2020). Astrocytes modulate neuronal activity and support cognitive functions in both human and mammalian species (Pereira and Furlan, 2010; Ferris et al., 2017). Activated astrocytes participate in cognitive impairment in multiple diseases (Santello et al., 2019; Calsolaro et al., 2021). HFD consumption-induced microglial activation and chronic inflammation may lead to astrocytes activation in the brain regions regulation of cognition. It’s reported that chronic consumption of high-fat-and-fructose diets increases the number and coverage of hippocampal reactive astrocytes associated with microglial morphological changes and reduced dendritic arborization, spine number, and synaptophysin content in CA1 in rats (Calvo-Ochoa et al., 2014). HFD also increases the reactivity and disturbs the function of hippocampal astrocytes in mice (Tsai et al., 2018). Moreover, astrocytes regulate the function of BBB. Reactive astrocyte-derived factors increase the permeability of BBB and lead to BBB disruption (Michinaga and Koyama, 2019). High-saturated-fat and cholesterol diet also impairs hippocampal BBB integrity in rats, which may be associated with increases microglial and astrocytes activation in this brain region (Freeman and Granholm, 2012; Yeh et al., 2015; Han et al., 2020). Additionally, HFD feeding disrupts BBB through induction of cerebrovascular and endothelial dysfunction (Li et al., 2013; Nguyen et al., 2014; Buie et al., 2019).

The excessive BBB permeability promotes the infiltration of peripheral immune cells and blood-derived molecules into the brain parenchyma (Freeman and Granholm, 2012; Michinaga and Koyama, 2019). Recruitment of peripheral immune cells into the brain contributes to the inflammatory response in HFD fed mice (Buckman et al., 2014). It’s known that the infiltrated immune cells and factors induce microglial and astrocytes activation and are associated with inflammation, neuronal damage, and cognitive decline (Puntener et al., 2012; Widmann and Heneka, 2014; Laurent et al., 2017). Moreover, HFD consumption leads to chronic low-grade peripheral inflammation, which has been regarded as a risk factor for cognitive decline (Saltiel and Olefsky, 2017; Duan et al., 2018; Tan and Norhaizan, 2019). Emerging evidence showed that the peripheral or systemic inflammation produced from adipose tissue and gut triggers microglial activation and neuroinflammation and exacerbates cognitive deficits in obese individuals (Chunchai et al., 2018; Guo et al., 2020; Lee and Yau, 2021). Reducing the imbalance of gut microbiota induced by HFD consumption alleviates hippocampal oxidative stress and microglial activation and restores cognitive function (Chunchai et al., 2018). Interestingly, a study showed that switching the HFD to a low-fat diet for 2 months reduces microglial activation and rescues the synaptic plasticity and cognition in adult obese mice (Hao et al., 2016).

## Conclusion

In conclusion, this review has summarized the recent studies in the understanding of microglial function in governing brain circuits mainly associated with cognition and energy homeostasis. The prevailing view of microglia as phagocytic cells eliminating synapses, dead or apoptotic cells, and cellular debris may be overly simplified. Indeed, microglia have a critical role in the synaptic formation, reorganization, appropriate maturation, and neurogenesis, by direct contacting, releasing soluble factors, engulfment of synaptic structures, or multiple microglia-neuronal signaling pathways during brain circuits remolding; this process occurs throughout life to adapt to the changing brain microenvironment. Microglia are necessary for the proper development and maintenance of hippocampal and hypothalamic neuronal circuits, however, microglial dysfunction in these brain regions leads to cognitive decline and obesity, respectively. Furthermore, microglial activation is involved in both HFD induced obesity and obesity-associated cognitive impairment (Figure 1). But how the HFD consumption leads to hypothalamic microglial activation and results in hippocampal microglial dysfunction are still unclear. Since the heterogeneity of microglia, our knowledge of how microglia shape neuronal circuitries is still superficial, especially under HFD conditions. A more detailed understanding of the mechanism is needed, such as the molecules that impact microglial functions and the complex interplay between microglia and neurons, as well as periphery signals and microglia. Integrated modulation of microglial function might be an effective strategy to prevent neurodegeneration and obesity.

## Author Contributions

X-LW and LL conceived the work and wrote the manuscript. Both authors contributed to the article and approved the submitted version.

## Conflict of Interest

The authors declare that the research was conducted in the absence of any commercial or financial relationships that could be construed as a potential conflict of interest.

## Publisher’s Note

All claims expressed in this article are solely those of the authors and do not necessarily represent those of their affiliated organizations, or those of the publisher, the editors and the reviewers. Any product that may be evaluated in this article, or claim that may be made by its manufacturer, is not guaranteed or endorsed by the publisher.

## References

[B1] Aarum J. Sandberg K. Haeberlein S. L. B. Persson M. A. A. (2003). Migration and differentiation of neural precursor cells can be directed by microglia. *Proc. Natl. Acad. Sci. USA.* 100:15983. 10.1073/pnas.2237050100 14668448 PMC307679

[B2] Abdalla M. M. (2017). Central and peripheral control of food intake. *Endocr. Regul.* 51:52. 10.1515/enr-2017-0006/j/enr.2017.51.issue-1/enr-2017-0006/enr-2017-0006.xml 28222022

[B3] Aloisi F. (2001). Immune function of microglia. *Glia* 36:165. 11596125 10.1002/glia.1106

[B4] Andre C. Guzman-Quevedo O. Rey C. Remus-Borel J. Clark S. Castellanos-Jankiewicz A. (2017). Inhibiting microglia expansion prevents diet-induced hypothalamic and peripheral inflammation. *Diabetes* 66:908. 10.2337/db16-0586db16-0586 27903745

[B5] Appel J. R. Ye S. Tang F. Sun D. Zhang H. Mei L. (2018). Increased microglial activity, impaired adult hippocampal neurogenesis, and depressive-like behavior in microglial VPS35-depleted mice. *J. Neurosci.* 38:5949. 10.1523/JNEUROSCI.3621-17.2018 29853629 PMC6021995

[B6] Araque A. Carmignoto G. Haydon P. G. Oliet S. H. Robitaille R. Volterra A. (2014). Gliotransmitters travel in time and space. *Neuron* 81:728. 10.1016/j.neuron.2014.02.007S0896-6273(14)00105-6 24559669 PMC4107238

[B7] Areschoug T. Gordon S. (2009). Scavenger receptors: role in innate immunity and microbial pathogenesis. *Cell. Microbiol.* 11:1160. 10.1111/j.1462-5822.2009.01326.x 19388903

[B8] Argaw A. T. Asp L. Zhang J. Navrazhina K. Pham T. Mariani J. N. (2012). Astrocyte-derived VEGF-A drives blood-brain barrier disruption in CNS inflammatory disease. *J. Clin. Invest.* 122:2454. 10.1172/JCI6084260842 22653056 PMC3386814

[B9] Ashwell K. (1990). Microglia and cell death in the developing mouse cerebellum. *Brain Res. Dev. Brain Res.* 55:219. 2253324 10.1016/0165-3806(90)90203-b

[B10] Avalos Y. Kerr B. Maliqueo M. Dorfman M. (2018). Cell and molecular mechanisms behind diet-induced hypothalamic inflammation and obesity. *J. Neuroendocrinol.* 30:e12598. 10.1111/jne.12598 29645315

[B11] Avital A. Goshen I. Kamsler A. Segal M. Iverfeldt K. Richter-Levin G. (2003). Impaired interleukin-1 signaling is associated with deficits in hippocampal memory processes and neural plasticity. *Hippocampus* 13:826. 10.1002/hipo.10135 14620878

[B12] Ayata P. Badimon A. Strasburger H. J. Duff M. K. Montgomery S. E. Loh Y. E. (2018). Epigenetic regulation of brain region-specific microglia clearance activity. *Nat. Neurosci.* 21:1049. 10.1038/s41593-018-0192-310.1038/s41593-018-0192-3 30038282 PMC6090564

[B13] Ayoub A. E. Salm A. K. (2003). Increased morphological diversity of microglia in the activated hypothalamic supraoptic nucleus. *J. Neurosci.* 23:7759. 12944504 10.1523/JNEUROSCI.23-21-07759.2003 PMC6740605

[B14] Baufeld C. Osterloh A. Prokop S. Miller K. R. Heppner F. L. (2016). High-fat diet-induced brain region-specific phenotypic spectrum of CNS resident microglia. *Acta Neuropathol.* 132:361. 10.1007/s00401-016-1595-410.1007/s00401-016-1595-4 27393312 PMC4992033

[B15] Bellinger F. P. Madamba S. Siggins G. R. (1993). Interleukin 1 beta inhibits synaptic strength and long-term potentiation in the rat CA1 hippocampus. *Brain Res* 628:227. 10.1016/0006-8993(93)90959-q 8313151

[B16] Bisht K. Sharma K. P. Lecours C. Sanchez M. G. El Hajj H. Milior G. (2016). Dark microglia: A new phenotype predominantly associated with pathological states. *Glia* 64:826. 10.1002/glia.22966 26847266 PMC4949554

[B17] Bocarsly M. E. Fasolino M. Kane G. A. LaMarca E. A. Kirschen G. W. Karatsoreos I. N. (2015). Obesity diminishes synaptic markers, alters microglial morphology, and impairs cognitive function. *Proc. Natl. Acad. Sci. USA.* 112:15731. 10.1073/pnas.1511593112 26644559 PMC4697408

[B18] Bolos M. Perea J. R. Terreros-Roncal J. Pallas-Bazarra N. Jurado-Arjona J. Avila J. (2018). Absence of microglial CX3CR1 impairs the synaptic integration of adult-born hippocampal granule neurons. *Brain Behav. Immun.* 68:76. 10.1016/j.bbi.2017.10.002 29017970

[B19] Bray G. A. Smith S. R. DeJonge L. de Souza R. Rood J. Champagne C. M. (2012). Effect of diet composition on energy expenditure during weight loss: the POUNDS LOST Study. *Int J Obes (Lond)* 36:448. 10.1038/ijo.2011.173ijo2011173 21946707 PMC3289771

[B20] Brown G. C. Neher J. J. (2014). Microglial phagocytosis of live neurons. *Nat. Rev. Neurosci.* 15:209. 10.1038/nrn3710 24646669

[B21] Bruce-Keller A. J. White C. L. Gupta S. Knight A. G. Pistell P. J. Ingram D. K. (2010). NOX activity in brain aging: Exacerbation by high fat diet. *Free Radical. Bio. Med.* 49:22. 10.1016/j.freeradbiomed.2010.03.006 20347034 PMC2875353

[B22] Buckman L. B. Hasty A. H. Flaherty D. K. Buckman C. T. Thompson M. M. Matlock B. K. (2014). Obesity induced by a high-fat diet is associated with increased immune cell entry into the central nervous system. *Brain Behav. Immun.* 35:33. 10.1016/j.bbi.2013.06.007S0889-1591(13)00231-6 23831150 PMC3858467

[B23] Buie J. J. Watson L. S. Smith C. J. Sims-Robinson C. (2019). Obesity-related cognitive impairment: The role of endothelial dysfunction. *Neurobiol. Dis.* 132:104580. 10.1016/j.nbd.2019.104580 31454547 PMC6834913

[B24] Butovsky O. Ziv Y. Schwartz A. Landa G. Talpalar A. E. Pluchino S. (2006). Microglia activated by IL-4 or IFN-gamma differentially induce neurogenesis and oligodendrogenesis from adult stem/progenitor cells. *Mol. Cell. Neurosci.* 31:149. 10.1016/j.mcn.2005.10.006 16297637

[B25] Calsolaro V. Matthews P. M. Donat C. K. Livingston N. R. Femminella G. D. Guedes S. S. (2021). Astrocyte reactivity with late-onset cognitive impairment assessed in vivo using (11)C-BU99008 PET and its relationship with amyloid load. *Mol. Psychiatry* 10.1038/s41380-021-01193-z10.1038/s41380-021-01193-z [Online ahead of print]. PMC8758500 34267329

[B26] Calvo-Ochoa E. Hernandez-Ortega K. Ferrera P. Morimoto S. Arias C. (2014). Short-term high-fat-and-fructose feeding produces insulin signaling alterations accompanied by neurite and synaptic reduction and astroglial activation in the rat hippocampus. *J. Cereb. Blood Flow Metab.* 34:1001. 10.1038/jcbfm.2014.48jcbfm201448 24667917 PMC4050245

[B27] Caroni P. Chowdhury A. Lahr M. (2014). Synapse rearrangements upon learning: from divergent-sparse connectivity to dedicated sub-circuits. *Trends Neurosci.* 37:604. 10.1016/j.tins.2014.08.011 25257207

[B28] Cheray M. Joseph B. (2018). Epigenetics control microglia plasticity. *Front. Cell. Neurosci.* 12:243. 10.3389/Fncel.2018.00243 30123114 PMC6085560

[B29] Chunchai T. Thunapong W. Yasom S. Wanchai K. Eaimworawuthikul S. Metzler G. (2018). Decreased microglial activation through gut-brain axis by prebiotics, probiotics, or synbiotics effectively restored cognitive function in obese-insulin resistant rats. *J. Neuroinflamm.* 15:11. 10.1186/s12974-018-1055-2 PMC5761137 29316965

[B30] Collingridge G. L. Peineau S. (2014). Strippers reveal their depressing secrets: removing AMPA receptors. *Neuron* 82:3. 10.1016/j.neuron.2014.03.019 24698263

[B31] Colonna M. Butovsky O. (2017). Microglia function in the central nervous system during health and neurodegeneration. *Annu. Rev. Immunol.* 35:441. 10.1146/annurev-immunol-051116-052358 28226226 PMC8167938

[B32] Cope E. C. LaMarca E. A. Monari P. K. Olson L. B. Martinez S. Zych A. D. (2018). Microglia play an active role in obesity-associated cognitive decline. *J. Neurosci.* 38:8889. 10.1523/Jneurosci.0789-18.2018 30201764 PMC6181311

[B33] Coull J. A. M. Beggs S. Boudreau D. Boivin D. Tsuda M. Inoue K. (2005). BDNF from microglia causes the shift in neuronal anion gradient underlying neuropathic pain. *Nature* 438:1017. 10.1038/nature04223 16355225

[B34] Cunningham C. L. Martinez-Cerdeno V. Noctor S. C. (2013). Microglia regulate the number of neural precursor cells in the developing cerebral cortex. *J. Neurosci.* 33:4216. 10.1523/JNEUROSCI.3441-12.2013 23467340 PMC3711552

[B35] Damisah E. C. Hill R. A. Rai A. Chen F. Y. Rothlin C. V. Ghosh S. (2020). Astrocytes and microglia play orchestrated roles and respect phagocytic territories during neuronal corpse removal in vivo. *Sci. Adv.* 6:3239. 10.1126/sciadv.aba3239 32637606 PMC7319765

[B36] Das R. Chinnathambi S. (2019). Microglial priming of antigen presentation and adaptive stimulation in Alzheimer’s disease. *Cell Mol Life Sci.* 76 3681–3694. 10.1007/s00018-019-03132-2 31093687 PMC11105582

[B37] Davalos D. Grutzendler J. Yang G. Kim J. V. Zuo Y. Jung S. (2005). ATP mediates rapid microglial response to local brain injury in vivo. *Nat. Neurosci.* 8:752. 10.1038/nn1472 15895084

[B38] de Miranda A. S. Zhang C. J. Katsumoto A. Teixeira A. L. (2017). Hippocampal adult neurogenesis: Does the immune system matter? *J. Neurol. Sci.* 372:482. 10.1016/j.jns.2016.10.052 27838002

[B39] De Souza C. T. Araujo E. P. Bordin S. Ashimine R. Zollner R. L. Boschero A. C. (2005). Consumption of a fat-rich diet activates a proinflammatory response and induces insulin resistance in the hypothalamus. *Endocrinology* 146:4192. 10.1210/en.2004-1520 16002529

[B40] Di Cesare M. Bentham J. Stevens G. A. Zhou B. Danaei G. Lu Y. (2016). Trends in adult body-mass index in 200 countries from 1975 to 2014: a pooled analysis of 1698 population-based measurement studies with 19.2 million participants. *Lancet* 387:1377. 10.1016/s0140-6736(16)30054-x 27115820 PMC7615134

[B41] Djogo T. Robins S. C. Schneider S. Kryzskaya D. Liu X. Mingay A. (2016). Adult NG2-glia are required for median eminence-mediated leptin sensing and body weight control. *Cell. Metab.* 23:797. 10.1016/j.cmet.2016.04.013S1550-4131(16)30164-4 27166944

[B42] Douglass J. D. Dorfman M. D. Fasnacht R. Shaffer L. D. Thaler J. P. (2017). Astrocyte IKKbeta/NF-kappaB signaling is required for diet-induced obesity and hypothalamic inflammation. *Mol. Metab.* 6:366. 10.1016/j.molmet.2017.01.010 28377875 PMC5369266

[B43] Duan Y. Zeng L. Zheng C. Song B. Li F. Kong X. (2018). Inflammatory links between high fat diets and diseases. *Front. Immunol.* 9:2649. 10.3389/fimmu.2018.02649 30483273 PMC6243058

[B44] Edison P. (2020). Neuroinflammation, microglial activation, and glucose metabolism in neurodegenerative diseases. *Int. Rev. Neurobiol.* 154:325. 10.1016/bs.irn.2020.03.017 32739010

[B45] Edwards L. M. Murray A. J. Holloway C. J. Carter E. E. Kemp G. J. Codreanu I. (2011). Short-term consumption of a high-fat diet impairs whole-body efficiency and cognitive function in sedentary men. *FASEB J.* 25:1088. 10.1096/fj.10-171983 21106937

[B46] El Khoury J. Toft M. Hickman S. E. Means T. K. Terada K. Geula C. (2007). Ccr2 deficiency impairs microglial accumulation and accelerates progression of Alzheimer-like disease. *Nat. Med.* 13:432. 10.1038/nm1555 17351623

[B47] Ellacott K. L. Cone R. D. (2004). The central melanocortin system and the integration of short- and long-term regulators of energy homeostasis. *Recent Prog. Horm. Res.* 59:395. 14749511 10.1210/rp.59.1.395

[B48] Elmore M. R. P. Hohsfield L. A. Kramar E. A. Soreq L. Lee R. J. Pham S. T. (2018). Replacement of microglia in the aged brain reverses cognitive, synaptic, and neuronal deficits in mice. *Aging Cell.* 17:e12832. 10.1111/acel.12832 30276955 PMC6260908

[B49] Elmore M. R. P. Najafi A. R. Koike M. A. Dagher N. N. Spangenberg E. E. Rice R. A. (2014). Colony-stimulating factor 1 receptor signaling is necessary for microglia viability, unmasking a microglia progenitor cell in the adult brain. *Neuron* 82:380. 10.1016/j.neuron.2014.02.040 24742461 PMC4161285

[B50] Engel D. F. Bobbo V. C. D. Solon C. S. Nogueira G. A. Moura-Assis A. Mendes N. F. (2020). Activation of GPR40 induces hypothalamic neurogenesis through p38- and BDNF-dependent mechanisms. *Sci. Rep.* 10:11047. 10.1038/s41598-020-68110-210.1038/s41598-020-68110-2 32632088 PMC7338363

[B51] Ernst A. Alkass K. Bernard S. Salehpour M. Perl S. Tisdale J. (2014). Neurogenesis in the striatum of the adult human brain. *Cell* 156:1072. 10.1016/j.cell.2014.01.044 24561062

[B52] Fakhoury M. (2018). Microglia and astrocytes in Alzheimer’s Disease: implications for therapy. *Curr. Neuropharmacol.* 16:508. 10.2174/1570159x15666170720095240 28730967 PMC5997862

[B53] Fernandez-Arjona M. D. M. Grondona J. M. Granados-Duran P. Fernandez-Llebrez P. Lopez-Avalos M. D. (2017). Microglia morphological categorization in a rat model of neuroinflammation by hierarchical cluster and principal components analysis. *Front. Cell. Neurosci.* 11:235. 10.3389/fncel.2017.00235 28848398 PMC5550745

[B54] Ferris H. A. Perry R. J. Moreira G. V. Shulman G. I. Horton J. D. Kahn C. R. (2017). Loss of astrocyte cholesterol synthesis disrupts neuronal function and alters whole-body metabolism. *Proc. Natl. Acad. Sci. U S A* 114:1189. 10.1073/pnas.16205061141620506114 28096339 PMC5293102

[B55] Filipello F. Morini R. Corradini I. Zerbi V. Canzi A. Michalski B. (2018). The microglial innate immune receptor TREM2 is required for synapse elimination and normal brain connectivity. *Immunity* 48:979. 10.1016/j.immuni.2018.04.016 29752066

[B56] Freeman L. R. Granholm A. C. (2012). Vascular changes in rat hippocampus following a high saturated fat and cholesterol diet. *J. Cereb. Blood Flow Metab.* 32:643. 10.1038/jcbfm.2011.168jcbfm2011168 22108721 PMC3318144

[B57] Fu R. Shen Q. Xu P. Luo J. J. Tang Y. (2014). Phagocytosis of microglia in the central nervous system diseases. *Mol. Neurobiol.* 49:1422. 10.1007/s12035-013-8620-6 24395130 PMC4012154

[B58] Fuger P. Hefendehl J. K. Veeraraghavalu K. Wendeln A. C. Schlosser C. Obermuller U. (2017). Microglia turnover with aging and in an Alzheimer’s model via long-term in vivo single-cell imaging. *Nat. Neurosci.* 20:1371. 10.1038/nn.4631 28846081

[B59] Gao Y. Ottaway N. Schriever S. C. Legutko B. Garcia-Caceres C. de la Fuente E. (2014). Hormones and diet, but not body weight, control hypothalamic microglial activity. *Glia* 62:17. 10.1002/glia.22580 24166765 PMC4213950

[B60] Gao Y. Vidal-Itriago A. Milanova I. Korpel N. L. Kalsbeek M. J. Tom R. Z. (2018). Deficiency of leptin receptor in myeloid cells disrupts hypothalamic metabolic circuits and causes body weight increase. *Mol. Metab.* 7:155. 29174000 10.1016/j.molmet.2017.11.003 PMC5784319

[B61] Gao Y. Q. Vidal-Itriago A. Kalsbeek M. J. Layritz C. Garcia-Caceres C. Tom R. Z. (2017). Lipoprotein lipase maintains microglial innate immunity in obesity. *Cell. Rep.* 20:3034. 10.1016/j.celrep.2017.09.008 28954222

[B62] Garcia-Caceres C. Balland E. Prevot V. Luquet S. Woods S. C. Koch M. (2019). Role of astrocytes, microglia, and tanycytes in brain control of systemic metabolism. *Nat. Neurosci.* 22:7. 10.1038/s41593-018-0286-y 30531847

[B63] Garcia-Prieto C. F. Hernandez-Nuno F. Rio D. D. Ruiz-Hurtado G. Aranguez I. Ruiz-Gayo M. (2015). High-fat diet induces endothelial dysfunction through a down-regulation of the endothelial AMPK-PI3K-Akt-eNOS pathway. *Mol. Nutr. Food Res.* 59:520. 10.1002/mnfr.201400539 25421217

[B64] Ginhoux F. Greter M. Leboeuf M. Nandi S. See P. Gokhan S. (2010). Fate mapping analysis reveals that adult microglia derive from primitive macrophages. *Science* 330:841. 10.1126/science.1194637 20966214 PMC3719181

[B65] Gonzalez-Scarano F. Baltuch G. (1999). Microglia as mediators of inflammatory and degenerative diseases. *Annu. Rev. Neurosci.* 22:219. 10.1146/annurev.neuro.22.1.219 10202538

[B66] Greenhalgh A. D. David S. Bennett F. C. (2020). Immune cell regulation of glia during CNS injury and disease. *Nat. Rev. Neurosci.* 21:139. 10.1038/s41583-020-0263-910.1038/s41583-020-0263-9 32042145

[B67] Guo D. H. Yamamoto M. Hernandez C. M. Khodadadi H. Baban B. Stranahan A. M. (2020). Visceral adipose NLRP3 impairs cognition in obesity via IL-1R1 on CX3CR1(+) cells. *J. Clin. Invest.* 130:1961. 10.1172/Jci126078 31935195 PMC7108893

[B68] Haan N. Goodman T. Najdi-Samiei A. Stratford C. M. Rice R. El Agha E. (2013). Fgf10-expressing tanycytes add new neurons to the appetite/energy-balance regulating centers of the postnatal and adult hypothalamus. *J. Neurosci.* 33:6170. 10.1523/JNEUROSCI.2437-12.201333/14/6170 23554498 PMC3736310

[B69] Hahn T. M. Breininger J. F. Baskin D. G. Schwartz M. W. (1998). Coexpression of Agrp and NPY in fasting-activated hypothalamic neurons. *Nat. Neurosci.* 1:271. 10.1038/1082 10195157

[B70] Hammond T. R. Dufort C. Dissing-Olesen L. Giera S. Young A. Wysoker A. (2019). Single-Cell RNA sequencing of microglia throughout the mouse lifespan and in the injured brain reveals complex cell-state changes. *Immunity* 50:253. 10.1016/j.immuni.2018.11.004 30471926 PMC6655561

[B71] Han X. Zhang T. Liu H. Mi Y. Gou X. (2020). Astrocyte senescence and Alzheimer’s disease: A review. *Front. Aging Neurosci.* 12:148. 10.3389/fnagi.2020.00148 32581763 PMC7297132

[B72] Hao S. Dey A. Yu X. L. Stranahan A. M. (2016). Dietary obesity reversibly induces synaptic stripping by microglia and impairs hippocampal plasticity. *Brain Behav. Immun.* 51:230. 10.1016/j.bbi.2015.08.023 26336035 PMC4679537

[B73] Haynes S. E. Hollopeter G. Yang G. Kurpius D. Dailey M. E. Gan W. B. (2006). The P2Y12 receptor regulates microglial activation by extracellular nucleotides. *Nat. Neurosci.* 9:1512. 10.1038/nn1805 17115040

[B74] Heneka M. T. Golenbock D. T. Latz E. (2015). Innate immunity in Alzheimer’s disease. *Nat. Immunol.* 16:229. 10.1038/ni.3102 25689443

[B75] Heyward F. D. Walton R. G. Carle M. S. Coleman M. A. Garvey W. T. Sweatt J. D. (2012). Adult mice maintained on a high-fat diet exhibit object location memory deficits and reduced hippocampal SIRT1 gene expression. *Neurobiol. Learn. Mem.* 98:25. 10.1016/j.nlm.2012.04.005 22542746 PMC3389577

[B76] Hickman S. Izzy S. Sen P. Morsett L. El Khoury J. (2018). Microglia in neurodegeneration. *Nat. Neurosci.* 21:1359. 10.1038/s41593-018-0242-x 30258234 PMC6817969

[B77] Hickman S. E. Kingery N. D. Ohsumi T. K. Borowsky M. L. Wang L. C. Means T. K. (2013). The microglial sensome revealed by direct RNA sequencing. *Nat. Neurosci.* 16:1896. 10.1038/nn.3554 24162652 PMC3840123

[B78] Hide I. Tanaka M. Inoue A. Nakajima K. Kohsaka S. Inoue K. (2000). Extracellular ATP triggers tumor necrosis factor-alpha release from rat microglia. *J. Neurochem.* 75:965. 10.1046/j.1471-4159.2000.0750965.x 10936177

[B79] Holloway C. J. Cochlin L. E. Emmanuel Y. Murray A. Codreanu I. Edwards L. M. (2011). A high-fat diet impairs cardiac high-energy phosphate metabolism and cognitive function in healthy human subjects. *Am. J. Clin. Nutr.* 93:748. 10.3945/ajcn.110.002758 21270386

[B80] Hong S. Beja-Glasser V. F. Nfonoyim B. M. Frouin A. Li S. M. Ramakrishnan S. (2016). Complement and microglia mediate early synapse loss in Alzheimer mouse models. *Science* 352:712. 10.1126/science.aad8373 27033548 PMC5094372

[B81] Horvath T. L. Sarman B. Garcia-Caceres C. Enriori P. J. Sotonyi P. Shanabrough M. (2010). Synaptic input organization of the melanocortin system predicts diet-induced hypothalamic reactive gliosis and obesity. *Proc. Natl. Acad. Sci. U S A* 107:14875. 10.1073/pnas.10042821071004282107 20679202 PMC2930476

[B82] Hoshiko M. Arnoux I. Avignone E. Yamamoto N. Audinat E. (2012). Deficiency of the microglial receptor CX3CR1 impairs postnatal functional development of thalamocortical synapses in the barrel cortex. *J. Neurosci.* 32:15106. 10.1523/JNEUROSCI.1167-12.2012 23100431 PMC6704837

[B83] Hua J. Y. Smith S. J. (2004). Neural activity and the dynamics of central nervous system development. *Nat. Neurosci.* 7:327. 10.1038/nn1218 15048120

[B84] Huttenlocher P. R. (1979). Synaptic density in human frontal cortex - developmental changes and effects of aging. *Brain Res.* 163:195. 10.1016/0006-8993(79)90349-4 427544

[B85] Ikegami A. Haruwaka K. Wake H. (2019). Microglia: Lifelong modulator of neural circuits. *Neuropathology* 39:173. 10.1111/neup.12560 31131941

[B86] Jais A. Bruning J. C. (2017). Hypothalamic inflammation in obesity and metabolic disease. *J. Clin. Invest.* 127:24. 10.1172/JCI88878 28045396 PMC5199695

[B87] Jeong M. Y. Jang H. M. Kim D. H. (2019). High-fat diet causes psychiatric disorders in mice by increasing *Proteobacteria* population. *Neurosci. Lett.* 698:51. 10.1016/j.neulet.2019.01.006 30615977

[B88] Ji K. Akgul G. Wollmuth L. P. Tsirka S. E. (2013). Microglia actively regulate the number of functional synapses. *PLoS One* 8:e56293. 10.1371/journal.pone.0056293 23393609 PMC3564799

[B89] Jiang X. Nardelli J. (2016). Cellular and molecular introduction to brain development. *Neurobiol. Dis.* 92:3. 10.1016/j.nbd.2015.07.007 26184894 PMC4720585

[B90] Jin S. Kim K. K. Park B. S. Kim D. H. Jeong B. Kang D. (2020). Function of astrocyte MyD88 in high-fat-diet-induced hypothalamic inflammation. *J. Neuroinflamm.* 17:195. 10.1186/s12974-020-01846-w10.1186/s12974-020-01846-w PMC7304177 32560726

[B91] Kabaso D. Coskren P. J. Henry B. I. Hof P. R. Wearne S. L. (2009). The electrotonic structure of pyramidal neurons contributing to prefrontal cortical circuits in macaque monkeys is significantly altered in aging. *Cereb. Cortex* 19:2248. 10.1093/cercor/bhn242 19150923 PMC2742588

[B92] Keren-Shaul H. Spinrad A. Weiner A. Matcovitch-Natan O. Dvir-Szternfeld R. Ulland T. K. (2017). A unique microglia type associated with restricting development of Alzheimer’s Disease. *Cell* 169:1276. 10.1016/j.cell.2017.05.018 28602351

[B93] Kim C. S. Kwon Y. Choe S. Y. Hong S. M. Yoo H. Goto T. (2015). Quercetin reduces obesity-induced hepatosteatosis by enhancing mitochondrial oxidative metabolism via heme oxygenase-1. *Nutr. Metab. (Lond).* 12:33. 10.1186/s12986-015-0030-5 26445592 PMC4595266

[B94] Kim J. D. Yoon N. A. Jin S. Diano S. (2019). Microglial UCP2 mediates inflammation and obesity induced by high-fat feeding. *Cell. Metab.* 30:952. 10.1016/j.cmet.2019.08.010 31495690 PMC7251564

[B95] Kim J. G. Suyama S. Koch M. Jin S. Argente-Arizon P. Argente J. (2014). Leptin signaling in astrocytes regulates hypothalamic neuronal circuits and feeding. *Nat. Neurosci.* 17:908. 10.1038/nn.3725nn.3725 24880214 PMC4113214

[B96] Kirkley K. S. Popichak K. A. Afzali M. F. Legare M. E. Tjalkens R. B. (2017). Microglia amplify inflammatory activation of astrocytes in manganese neurotoxicity. *J. Neuroinflamm.* 14:99. 10.1186/s12974-017-0871-010.1186/s12974-017-0871-0 PMC5418760 28476157

[B97] Koch M. Varela L. Kim J. G. Kim J. D. Hernandez-Nuno F. Simonds S. E. (2015). Hypothalamic POMC neurons promote cannabinoid-induced feeding. *Nature* 519:45. 10.1038/nature14260nature14260 25707796 PMC4496586

[B98] Kokoeva M. V. Yin H. Flier J. S. (2005). Neurogenesis in the hypothalamus of adult mice: potential role in energy balance. *Science* 310:679. 10.1126/science.1115360 16254185

[B99] Krady J. K. Lin H. W. Liberto C. M. Basu A. Kremlev S. G. Levison S. W. (2008). Ciliary neurotrophic factor and interleukin-6 differentially activate microglia. *J. Neurosci. Res.* 86:1538. 10.1002/jnr.21620 18214991

[B100] Krashes M. J. Koda S. Ye C. Rogan S. C. Adams A. C. Cusher D. S. (2011). Rapid, reversible activation of AgRP neurons drives feeding behavior in mice. *J. Clin. Invest.* 121:1424. 10.1172/JCI46229 21364278 PMC3069789

[B101] Lau J. Herzog H. (2014). CART in the regulation of appetite and energy homeostasis. *Front. Neurosci.* 8:313. 10.3389/fnins.2014.00313 25352770 PMC4195273

[B102] Laurent C. Dorothee G. Hunot S. Martin E. Monnet Y. Duchamp M. (2017). Hippocampal T cell infiltration promotes neuroinflammation and cognitive decline in a mouse model of tauopathy. *Brain.* 140:184. 10.1093/brain/aww270 27818384 PMC5382942

[B103] Le N. H. Kim C. S. Park T. Park J. H. Y. Sung K. Lee D. G. (2014). Quercetin protects against obesity-induced skeletal muscle inflammation and atrophy. *Mediat. Inflamm.* 2014:834294. 10.1155/2014/834294 25614714 PMC4295595

[B104] Le Thuc O. Stobbe K. Cansell C. Nahon J. L. Blondeau N. Rovere C. (2017). Hypothalamic inflammation and energy balance disruptions: spotlight on chemokines. *Front. Endocrinol. (Lausanne).* 8:197. 10.3389/fendo.2017.00197 28855891 PMC5557773

[B105] Lechan R. M. Fekete C. (2006). The TRH neuron: a hypothalamic integrator of energy metabolism. *Prog. Brain Res.* 153:209. 10.1016/S0079-6123(06)53012-2 16876577

[B106] Lee C. H. Kim H. J. Lee Y. S. Kang G. M. Lim H. S. Lee S. H. (2018). Hypothalamic macrophage inducible nitric oxide synthase mediates obesity-associated hypothalamic inflammation. *Cell. Rep.* 25:934. 10.1016/j.celrep.2018.09.070 30355499 PMC6284237

[B107] Lee T. H. Y. Yau S. Y. (2021). From obesity to hippocampal neurodegeneration: pathogenesis and non-pharmacological interventions. *Int. J. Mol. Sci.* 22:201. 10.3390/ijms22010201 PMC7796248 33379163

[B108] Lemke G. (2013). Biology of the TAM receptors. *Cold Spring Harb. Perspect. Biol.* 5:a009076. 10.1101/cshperspect.a009076 24186067 PMC3809585

[B109] Li Q. Cheng Z. Zhou L. Darmanis S. Neff N. F. Okamoto J. (2019). Developmental heterogeneity of microglia and brain myeloid cells revealed by deep single-cell RNA sequencing. *Neuron* 101:207. 30606613 10.1016/j.neuron.2018.12.006 PMC6336504

[B110] Li W. Prakash R. Chawla D. Du W. Didion S. P. Filosa J. A. (2013). Early effects of high-fat diet on neurovascular function and focal ischemic brain injury. *Am. J. Physiol. Regul. Integr. Comp. Physiol.* 304:R1001. 10.1152/ajpregu.00523.2012ajpregu.00523.2012 23576615 PMC3680754

[B111] Liddelow S. A. Guttenplan K. A. Larke L. E. C. Bennett F. C. Bohlen C. J. Schirmer L. (2017). Neurotoxic reactive astrocytes are induced by activated microglia. *Nature* 541:481. 10.1038/nature21029 28099414 PMC5404890

[B112] Lim S. H. Park E. You B. Jung Y. Park A. R. Park S. G. (2013). Neuronal synapse formation induced by microglia and interleukin 10. *PLoS One* 8:e81218. 10.1371/journal.pone.0081218 24278397 PMC3838367

[B113] Lin H. W. Jain M. R. Li H. Levison S. W. (2009). Ciliary neurotrophic factor (CNTF) plus soluble CNTF receptor alpha increases cyclooxygenase-2 expression, PGE2 release and interferon-gamma-induced CD40 in murine microglia. *J. Neuroinflamm.* 6:7. 10.1186/1742-2094-6-71742-2094-6-7 PMC2660310 19267906

[B114] Lucin K. M. O’Brien C. E. Bieri G. Czirr E. Mosher K. I. Abbey R. J. (2013). Microglial beclin 1 regulates retromer trafficking and phagocytosis and is impaired in Alzheimer’s Disease. *Neuron* 79:873. 10.1016/j.neuron.2013.06.046 24012002 PMC3779465

[B115] Luquet S. Perez F. A. Hnasko T. S. Palmiter R. D. (2005). NPY/AgRP neurons are essential for feeding in adult mice but can be ablated in neonates. *Science* 310:683. 10.1126/science.1115524 16254186

[B116] Maldonado-Ruiz R. Montalvo-Martinez L. Fuentes-Mera L. Camacho A. (2017). Microglia activation due to obesity programs metabolic failure leading to type two diabetes. *Nutr Diabetes* 7:e254. 10.1038/nutd.2017.10 28319103 PMC5380893

[B117] Mallya A. P. Wang H. D. Lee H. N. R. Deutch A. Y. (2019). Microglial pruning of synapses in the prefrontal cortex during adolescence. *Cereb. Cortex* 29:1634. 10.1093/cercor/bhy061 29668872 PMC6418387

[B118] Mangold C. A. Wronowski B. Du M. Masser D. R. Hadad N. Bixler G. V. (2017). Sexually divergent induction of microglial-associated neuroinflammation with hippocampal aging. *J. Neuroinflamm.* 14:141. 10.1186/S12974-017-0920-8 28732515 PMC5521082

[B119] Margueron R. Reinberg D. (2011). The polycomb complex PRC2 and its mark in life. *Nature* 469:343. 10.1038/nature09784 21248841 PMC3760771

[B120] Masser D. R. Bixler G. V. Brucklacher R. M. Yan H. Giles C. B. Wren J. D. (2014). Hippocampal subregions exhibit both distinct and shared transcriptomic responses to aging and nonneurodegenerative cognitive decline. *J. Gerontol. Biol.* 69:1311. 10.1093/gerona/glu091 24994846 PMC4271093

[B121] Masuda T. Sankowski R. Staszewski O. Bottcher C. Amann L. Sagar (2019). Spatial and temporal heterogeneity of mouse and human microglia at single-cell resolution. *Nature* 566:388. 10.1038/s41586-019-0924-x10.1038/s41586-019-0924-x 30760929

[B122] Masuda T. Sankowski R. Staszewski O. Prinz M. (2020). Microglia heterogeneity in the single-cell era. *Cell Rep.* 30:1271. 10.1016/j.celrep.2020.01.010 32023447

[B123] Mendes N. F. Jara C. P. Zanesco A. M. de Araujo E. P. (2021). Hypothalamic Microglial Heterogeneity and Signature under High Fat Diet-Induced Inflammation. *Int. J. Mol. Sci.* 22:2256. 10.3390/ijms22052256ijms22052256 33668314 PMC7956484

[B124] Michinaga S. Koyama Y. (2019). Dual roles of astrocyte-derived factors in regulation of blood-brain barrier function after brain damage. *Int. J. Mol. Sci.* 20:571. 10.3390/ijms20030571ijms20030571 PMC6387062 30699952

[B125] Mildner A. Schmidt H. Nitsche M. Merkler D. Hanisch U. K. Mack M. (2007). Microglia in the adult brain arise from Ly-6ChiCCR2+ monocytes only under defined host conditions. *Nat. Neurosci.* 10:1544. 10.1038/nn2015 18026096

[B126] Miyamoto A. Wake H. Ishikawa A. W. Eto K. Shibata K. Murakoshi H. (2016). Microglia contact induces synapse formation in developing somatosensory cortex. *Nat. Commun.* 7:12540. 10.1038/ncomms12540 27558646 PMC5007295

[B127] Mody M. Cao Y. X. Cui Z. Z. Tay K. Y. Shyong A. Shimizu E. (2001). Genome-wide gene expression profiles of the developing mouse hippocampus. *Proc. Natl. Acad. Sci. U S A.* 98:8862. 10.1073/pnas.141244998 11438693 PMC37526

[B128] Moraes J. C. Coope A. Morari J. Cintra D. E. Roman E. A. Pauli J. R. (2009). High-fat diet induces apoptosis of hypothalamic neurons. *PLoS One* 4:e5045. 10.1371/journal.pone.0005045 19340313 PMC2661137

[B129] Morgan S. C. Taylor D. L. Pocock J. M. (2004). Microglia release activators of neuronal proliferation mediated by activation of mitogen-activated protein kinase, phosphatidylinositol-3-kinase/Akt and delta-Notch signalling cascades. *J. Neurochem.* 90:89. 10.1111/j.1471-4159.2004.02461.x 15198670

[B130] Morris M. J. Beilharz J. E. Maniam J. Reichelt A. C. Westbrook R. F. (2015). Why is obesity such a problem in the 21st century? The intersection of palatable food, cues and reward pathways, stress, and cognition. *Neurosci. Biobehav. R.* 58:36. 10.1016/j.neubiorev.2014.12.002 25496905

[B131] Morton G. J. Cummings D. E. Baskin D. G. Barsh G. S. Schwartz M. W. (2006). Central nervous system control of food intake and body weight. *Nature* 443:289. 10.1038/nature05026 16988703

[B132] Mountjoy K. G. (2010). Functions for pro-opiomelanocortin-derived peptides in obesity and diabetes. *Biochem. J.* 428 305. 10.1042/BJ20091957 20504281

[B133] Murray A. J. Knight N. S. Cochlin L. E. McAleese S. Deacon R. M. J. Rawlins J. N. P. (2009). Deterioration of physical performance and cognitive function in rats with short-term high-fat feeding. *FASEB J.* 23:4353. 10.1096/fj.09-139691 19667117

[B134] Myers M. G. Jr. Olson D. P. (2012). Central nervous system control of metabolism. *Nature* 491:357. 10.1038/nature11705nature11705 23151578

[B135] Nakata M. Yamamoto S. Okada T. Yada T. (2017). AAV-mediated IL-10 gene transfer counteracts inflammation in the hypothalamic arcuate nucleus and obesity induced by high-fat diet. *Neuropeptides* 62:87. 10.1016/j.npep.2016.11.009 27939689

[B136] Nguyen J. C. Killcross A. S. Jenkins T. A. (2014). Obesity and cognitive decline: role of inflammation and vascular changes. *Front. Neurosci.* 8:375. 10.3389/fnins.2014.00375 25477778 PMC4237034

[B137] Nimmerjahn A. Kirchhoff F. Helmchen F. (2005). Resting microglial cells are highly dynamic surveillants of brain parenchyma in vivo. *Science* 308:1314. 10.1126/science.1110647 15831717

[B138] Olah M. Biber K. Vinet J. Boddeke H. W. (2011). Microglia phenotype diversity. *CNS Neurol. Disord. Drug Targets* 10:108. 21143141 10.2174/187152711794488575

[B139] Orihuela R. McPherson C. A. Harry G. J. (2016). Microglial M1/M2 polarization and metabolic states. *Br. J. Pharmacol.* 173:649. 10.1111/bph.13139 25800044 PMC4742299

[B140] Palkovits M. (2003). Hypothalamic regulation of food intake. *Ideggyogy Sz.* 56:288. 14608950

[B141] Pannacciulli N. Del Parigi A. Chen K. W. Le D. S. N. T. Reiman E. M. Tataranni P. A. (2006). Brain abnormalities in human obesity: A voxel-based morphometric study. *Neuroimage* 31:1419. 10.1016/j.neuroimage.2006.01.047 16545583

[B142] Paolicelli R. C. Bolasco G. Pagani F. Maggi L. Scianni M. Panzanelli P. (2011). Synaptic pruning by microglia is necessary for normal brain development. *Science* 333:1456. 10.1126/science.1202529 21778362

[B143] Parkhurst C. N. Yang G. Ninan I. Savas J. N. Yates J. R. Lafaille J. J. (2013). Microglia promote learning-dependent synapse formation through brain-derived neurotrophic factor. *Cell* 155:1596. 10.1016/j.cell.2013.11.030 24360280 PMC4033691

[B144] Pedditizi E. Peters R. Beckett N. (2016). The risk of overweight/obesity in mid-life and late life for the development of dementia: a systematic review and meta-analysis of longitudinal studies. *Age Ageing.* 45:14. 10.1093/ageing/afv151 26764391

[B145] Pencea V. Bingaman K. D. Wiegand S. J. Luskin M. B. (2001). Infusion of brain-derived neurotrophic factor into the lateral ventricle of the adult rat leads to new neurons in the parenchyma of the striatum, septum, thalamus, and hypothalamus. *J. Neurosci.* 21:6706. 11517260 10.1523/JNEUROSCI.21-17-06706.2001 PMC6763082

[B146] Pereira A. Jr. Furlan F. A. (2010). Astrocytes and human cognition: modeling information integration and modulation of neuronal activity. *Prog. Neurobiol.* 92:405. 10.1016/j.pneurobio.2010.07.001S0301-0082(10)00132-2 20633599

[B147] Pierce A. A. Xu A. W. (2010). De novo neurogenesis in adult hypothalamus as a compensatory mechanism to regulate energy balance. *J. Neurosci.* 30:723. 10.1523/JNEUROSCI.2479-09.201030/2/723 20071537 PMC3080014

[B148] Pinto S. Roseberry A. G. Liu H. Diano S. Shanabrough M. Cai X. (2004). Rapid rewiring of arcuate nucleus feeding circuits by leptin. *Science* 304:110. 10.1126/science.1089459 15064421

[B149] Pistell P. J. Morrison C. D. Gupta S. Knight A. G. Keller J. N. Ingram D. K. (2010). Cognitive impairment following high fat diet consumption is associated with brain inflammation. *J. Neuroimmunol.* 219:25. 10.1016/j.jneuroim.2009.11.010 20004026 PMC2823983

[B150] Puntener U. Booth S. G. Perry V. H. Teeling J. L. (2012). Long-term impact of systemic bacterial infection on the cerebral vasculature and microglia. *J. Neuroinflamm.* 9:146. 10.1186/1742-2094-9-146 22738332 PMC3439352

[B151] Rajendran L. Paolicelli R. C. (2018). Microglia-mediated synapse loss in Alzheimer’s Disease. *J. Neurosci.* 38:2911. 10.1523/Jneurosci.1136-17.2017 29563239 PMC6596066

[B152] Raji C. A. Ho A. J. Parikshak N. N. Becker J. T. Lopez O. L. Kuller L. H. (2010). Brain structure and obesity. *Hum. Brain Mapp.* 31:353. 10.1002/hbm.20870 19662657 PMC2826530

[B153] Ramalho A. F. Bombassaro B. Dragano N. R. Solon C. Morari J. Fioravante M. (2018). Dietary fats promote functional and structural changes in the median eminence blood/spinal fluid interface-the protective role for BDNF. *J. Neuroinflamm.* 15:10. 10.1186/s12974-017-1046-810.1186/s12974-017-1046-8 PMC5761204 29316939

[B154] Ransohoff R. M. Stevens B. (2011). How many cell types does it take to wire a brain? *Science* 333:1391. 10.1126/science.1212112 21903801

[B155] Reis W. L. Yi C. X. Gao Y. Tschop M. H. Stern J. E. (2015). Brain innate immunity regulates hypothalamic arcuate neuronal activity and feeding behavior. *Endocrinology* 156:1303. 10.1210/en.2014-1849 25646713 PMC4399317

[B156] Reshef R. Kudryavitskaya E. Shani-Narkiss H. Isaacson B. Rimmerman N. Mizrahi A. (2017). The role of microglia and their CX3CR1 signaling in adult neurogenesis in the olfactory bulb. *Elife* 6:e30809. 10.7554/eLife.30809 29251592 PMC5734876

[B157] Rogers J. T. Morganti J. M. Bachstetter A. D. Hudson C. E. Peters M. M. Grimmig B. A. (2011). CX3CR1 deficiency leads to impairment of hippocampal cognitive function and synaptic plasticity. *J. Neurosci.* 31:16241. 10.1523/JNEUROSCI.3667-11.2011 22072675 PMC3236509

[B158] Rosin J. M. Vora S. R. Kurrasch D. M. (2018). Depletion of embryonic microglia using the CSF1R inhibitor PLX5622 has adverse sex-specific effects on mice, including accelerated weight gain, hyperactivity and anxiolytic-like behaviour. *Brain Behav. Immun.* 73:682. 10.1016/j.bbi.2018.07.023 30056204

[B159] Roumier A. Bechade C. Poncer J. C. Smalla K. H. Tomasello E. Vivier E. (2004). Impaired synaptic function in the microglial KARAP/DAP12-deficient mouse. *J. Neurosci.* 24:11421. 10.1523/JNEUROSCI.2251-04.2004 15601948 PMC6730361

[B160] Saltiel A. R. Olefsky J. M. (2017). Inflammatory mechanisms linking obesity and metabolic disease. *J. Clin. Invest.* 127 1–4. 10.1172/JCI92035 28045402 PMC5199709

[B161] Santello M. Toni N. Volterra A. (2019). Astrocyte function from information processing to cognition and cognitive impairment. *Nat. Neurosci.* 22:154. 10.1038/s41593-018-0325-810.1038/s41593-018-0325-8 30664773

[B162] Sarlus H. Heneka M. T. (2017). Microglia in Alzheimer’s disease. *J. Clin. Invest.* 127:3240. 10.1172/JCI90606 28862638 PMC5669553

[B163] Schafer D. P. Lehrman E. K. Kautzman A. G. Koyama R. Mardinly A. R. Yamasaki R. (2012). Microglia sculpt postnatal neural circuits in an activity and complement-dependent manner. *Neuron* 74:691. 10.1016/j.neuron.2012.03.026S0896-6273(12)00334-0 22632727 PMC3528177

[B164] Schafer D. P. Lehrman E. K. Stevens B. (2013). The “quad-partite” synapse: microglia-synapse interactions in the developing and mature CNS. *Glia* 61:24. 10.1002/glia.22389 22829357 PMC4082974

[B165] Schafer D. P. Stevens B. (2013). Phagocytic glial cells: sculpting synaptic circuits in the developing nervous system. *Curr. Opin. Neurobiol.* 23:1034. 10.1016/j.conb.2013.09.012 24157239 PMC3907950

[B166] Schwartz M. W. Woods S. C. Porte D. Jr. Seeley R. J. Baskin D. G. (2000). Central nervous system control of food intake. *Nature.* 404:661. 10.1038/35007534 10766253

[B167] Sellgren C. M. Gracias J. Watmuff B. Biag J. D. Thanos J. M. Whittredge P. B. (2019). Increased synapse elimination by microglia in schizophrenia patient-derived models of synaptic pruning. *Nat. Neurosci.* 22:374. 10.1038/s41593-018-0334-7 30718903 PMC6410571

[B168] Sellgren C. M. Sheridan S. D. Gracias J. Xuan D. Fu T. Perlis R. H. (2017). Patient-specific models of microglia-mediated engulfment of synapses and neural progenitors. *Mol. Psychiatry.* 22:170. 10.1038/mp.2016.220 27956744 PMC5285468

[B169] Sellner S. Paricio-Montesinos R. Spiess A. Masuch A. Erny D. Harsan L. A. (2016). Microglial CX3CR1 promotes adult neurogenesis by inhibiting Sirt 1/p65 signaling independent of CX3CL1. *Acta Neuropathol. Commun.* 4:102. 10.1186/s40478-016-0374-8 27639555 PMC5027111

[B170] Shigemoto-Mogami Y. Hoshikawa K. Goldman J. E. Sekino Y. Sato K. (2014). Microglia enhance neurogenesis and oligodendrogenesis in the early postnatal subventricular zone. *J. Neurosci.* 34:2231. 10.1523/JNEUROSCI.1619-13.2014 24501362 PMC3913870

[B171] Sierra A. Abiega O. Shahraz A. Neumann H. (2013). Janus-faced microglia: beneficial and detrimental consequences of microglial phagocytosis. *Front. Cell. Neurosci.* 7:6. 10.3389/fncel.2013.00006 23386811 PMC3558702

[B172] Sierra A. Encinas J. M. Deudero J. J. P. Chancey J. H. Enikolopov G. Overstreet-Wadiche L. S. (2010). Microglia shape adult hippocampal neurogenesis through apoptosis-coupled phagocytosis. *Cell Stem Cell.* 7:483. 10.1016/j.stem.2010.08.014 20887954 PMC4008496

[B173] Sierra A. Gottfried-Blackmore A. C. McEwen B. S. Bulloch K. (2007). Microglia derived from aging mice exhibit an altered inflammatory profile. *Glia* 55:412. 10.1002/glia.20468 17203473

[B174] Sipe G. O. Lowery R. L. Tremblay M. E. Kelly E. A. Lamantia C. E. Majewska A. K. (2016). Microglial P2Y12 is necessary for synaptic plasticity in mouse visual cortex. *Nat. Commun.* 7:10905. 10.1038/Ncomms10905 26948129 PMC4786684

[B175] Sousa C. Golebiewska A. Poovathingal S. K. Kaoma T. Pires-Afonso Y. Martina S. (2018). Single-cell transcriptomics reveals distinct inflammation-induced microglia signatures. *EMBO Rep.* 19:e46171. 10.15252/embr.201846171embr.201846171 30206190 PMC6216255

[B176] Spencer S. J. D’Angelo H. Soch A. Watkins L. R. Maier S. F. Barrientos R. M. (2017). High-fat diet and aging interact to produce neuroinflammation and impair hippocampal- and amygdalar-dependent memory. *Neurobiol. Aging* 58:88. 10.1016/j.neurobiolaging.2017.06.014 28719855 PMC5581696

[B177] Stephan A. H. Barres B. A. Stevens B. (2012). The complement system: an unexpected role in synaptic pruning during development and disease. *Annu. Rev. Neurosci.* 35:369. 10.1146/annurev-neuro-061010-113810 22715882

[B178] Stratoulias V. Venero J. L. Tremblay M. E. Joseph B. (2019). Microglial subtypes: diversity within the microglial community. *EMBO J.* 38:e101997. 10.15252/embj.2019101997 31373067 PMC6717890

[B179] Streit W. J. Walter S. A. Pennell N. A. (1999). Reactive microgliosis. *Prog. Neurobiol.* 57:563. 10221782 10.1016/s0301-0082(98)00069-0

[B180] Subhramanyam C. S. Wang C. Hu Q. Dheen S. T. (2019). Microglia-mediated neuroinflammation in neurodegenerative diseases. *Semin. Cell. Dev. Biol.* 94 112–120. 10.1016/j.semcdb.2019.05.004 31077796

[B181] Tan B. L. Norhaizan M. E. (2019). Effect of high-fat diets on oxidative stress, cellular inflammatory response and cognitive function. *Nutrients* 11:2579. 10.3390/nu11112579nu11112579 PMC6893649 31731503

[B182] Tan Y. L. Yuan Y. Tian L. (2020). Microglial regional heterogeneity and its role in the brain. *Mol. Psychiatry* 25:351. 10.1038/s41380-019-0609-810.1038/s41380-019-0609-8 31772305 PMC6974435

[B183] Tang Y. Le W. (2016). Differential roles of M1 and M2 microglia in neurodegenerative diseases. *Mol. Neurobiol.* 53:1181. 10.1007/s12035-014-9070-5 25598354

[B184] Thaler J. P. Yi C. X. Schur E. A. Guyenet S. J. Hwang B. H. Dietrich M. O. (2012). Obesity is associated with hypothalamic injury in rodents and humans. *J. Clin. Invest.* 122:153. 10.1172/JCI5966059660 22201683 PMC3248304

[B185] Tikka T. Fiebich B. L. Goldsteins G. Keinanen R. Koistinaho J. (2001). Minocycline, a tetracycline derivative, is neuroprotective against excitotoxicity by inhibiting activation and proliferation of microglia. *J. Neurosci.* 21:2580. 10.1523/Jneurosci.21-08-02580.2001 11306611 PMC6762519

[B186] Toda C. Santoro A. Kim J. D. Diano S. (2017). POMC neurons: from birth to death. *Annu. Rev. Physiol.* 79:209. 10.1146/annurev-physiol-022516-034110 28192062 PMC5669621

[B187] Tremblay M. E. Lowery R. L. Majewska A. K. (2010). Microglial interactions with synapses are modulated by visual experience. *PLoS Biol.* 8:e1000527. 10.1371/journal.pbio.1000527 21072242 PMC2970556

[B188] Tremblay M. E. Stevens B. Sierra A. Wake H. Bessis A. Nimmerjahn A. (2011). The role of microglia in the healthy brain. *J. Neurosci.* 31:16064. 10.1523/Jneurosci.4158-11.2011 22072657 PMC6633221

[B189] Tsai S. F. Wu H. T. Chen P. C. Chen Y. W. Yu M. Wang T. F. (2018). High-fat diet suppresses the astrocytic process arborization and downregulates the glial glutamate transporters in the hippocampus of mice. *Brain Res.* 1700:66. 10.1016/j.brainres.2018.07.017 30009766

[B190] Ueno M. Fujita Y. Tanaka T. Nakamura Y. Kikuta J. Ishii M. (2013). Layer V cortical neurons require microglial support for survival during postnatal development. *Nat. Neurosci.* 16:543. 10.1038/nn.3358 23525041

[B191] Urabe H. Kojima H. Chan L. Terashima T. Ogawa N. Katagi M. (2013). Haematopoietic cells produce BDNF and regulate appetite upon migration to the hypothalamus. *Nat. Commun.* 4:1526. 10.1038/ncomms2536 23443554 PMC3640364

[B192] Valcarcel-Ares M. N. Tucsek Z. Kiss T. Giles C. B. Tarantini S. Yabluchanskiy A. (2019). Obesity in aging exacerbates neuroinflammation, dysregulating synaptic function-related genes and altering eicosanoid synthesis in the mouse hippocampus: potential role in impaired synaptic plasticity and cognitive decline. *J. Gerontol. Biol.* 74:290. 10.1093/gerona/gly127 29893815 PMC6376091

[B193] Valdearcos M. Douglass J. D. Robblee M. M. Dorfman M. D. Stifler D. R. Bennett M. L. (2017). Microglial inflammatory signaling orchestrates the hypothalamic immune response to dietary excess and mediates obesity susceptibility. *Cell. Metab.* 26:185. 10.1016/j.cmet.2017.05.015 28683286 PMC5569901

[B194] Valdearcos M. Robblee M. M. Benjamin D. I. Nomura D. K. Xu A. W. Koliwad S. K. (2014). Microglia dictate the impact of saturated fat consumption on hypothalamic inflammation and neuronal function. *Cell. Rep.* 9:2124. 10.1016/j.celrep.2014.11.018S2211-1247(14)00972-3 25497089 PMC4617309

[B195] VanRyzin J. W. Marquardt A. E. Argue K. J. Vecchiarelli H. A. Ashton S. E. Arambula S. E. (2019). Microglial phagocytosis of newborn cells is induced by endocannabinoids and sculpts sex differences in juvenile rat social play. *Neuron* 102:435. 10.1016/j.neuron.2019.02.006 30827729 PMC8046232

[B196] Verney C. Monier A. Fallet-Bianco C. Gressens P. (2010). Early microglial colonization of the human forebrain and possible involvement in periventricular white-matter injury of preterm infants. *J. Anat.* 217:436. 10.1111/j.1469-7580.2010.01245.x 20557401 PMC2992419

[B197] Vezzani A. Viviani B. (2015). Neuromodulatory properties of inflammatory cytokines and their impact on neuronal excitability. *Neuropharmacology.* 96:70. 10.1016/j.neuropharm.2014.10.027 25445483

[B198] Vukovic J. Colditz M. J. Blackmore D. G. Ruitenberg M. J. Bartlett P. F. (2012). Microglia modulate hippocampal neural precursor activity in response to exercise and aging. *J. Neurosci.* 32:6435. 10.1523/JNEUROSCI.5925-11.2012 22573666 PMC6621117

[B199] Wake H. Moorhouse A. J. Jinno S. Kohsaka S. Nabekura J. (2009). Resting microglia directly monitor the functional state of synapses in vivo and determine the fate of ischemic terminals. *J. Neurosci.* 29:3974. 10.1523/JNEUROSCI.4363-08.2009 19339593 PMC6665392

[B200] Walker F. R. Beynon S. B. Jones K. A. Zhao Z. Kongsui R. Cairns M. (2014). Dynamic structural remodelling of microglia in health and disease: a review of the models, the signals and the mechanisms. *Brain Behav. Immun.* 37:1. 10.1016/j.bbi.2013.12.010 24412599

[B201] Wang C. Billington C. J. Levine A. S. Kotz C. M. (2000). Effect of CART in the hypothalamic paraventricular nucleus on feeding and uncoupling protein gene expression. *Neuroreport* 11:3251. 10.1097/00001756-200009280-00040 11043558

[B202] Wang C. Yue H. Hu Z. Shen Y. Ma J. Li J. (2020). Microglia mediate forgetting via complement-dependent synaptic elimination. *Science* 367:688. 10.1126/science.aaz2288367/6478/688 32029629

[B203] Wang X. L. Kooijman S. Gao Y. Tzeplaeff L. Cosquer B. Milanova I. (2021). Microglia-specific knock-down of Bmal1 improves memory and protects mice from high fat diet-induced obesity. *Mol. Psychiatry* 10.1038/s41380-021-01169-z10.1038/s41380-021-01169-z [Online ahead of print]. PMC8760060 34050326

[B204] Wang Y. Cella M. Mallinson K. Ulrich J. D. Young K. L. Robinette M. L. (2015). TREM2 lipid sensing sustains the microglial response in an Alzheimer’s disease model. *Cell* 160:1061. 10.1016/j.cell.2015.01.049S0092-8674(15)00127-0 25728668 PMC4477963

[B205] Weinhard L. di Bartolomei G. Bolasco G. Machado P. Schieber N. L. Neniskyte U. (2018). Microglia remodel synapses by presynaptic trogocytosis and spine head filopodia induction. *Nat. Commun.* 9:1228. 10.1038/s41467-018-03566-5 29581545 PMC5964317

[B206] Widmann C. N. Heneka M. T. (2014). Long-term cerebral consequences of sepsis. *Lancet Neurol.* 13:630. 10.1016/S1474-4422(14)70017-1S1474-4422(14)70017-1 24849863

[B207] Wlodarczyk A. Holtman I. R. Krueger M. Yogev N. Bruttger J. Khorooshi R. (2017). A novel microglial subset plays a key role in myelinogenesis in developing brain. *EMBO J.* 36:3292. 10.15252/embj.201696056embj.201696056 28963396 PMC5686552

[B208] Wu Y. Dissing-Olesen L. MacVicar B. A. Stevens B. (2015). Microglia: dynamic mediators of synapse development and plasticity. *Trends Immunol.* 36:605. 26431938 10.1016/j.it.2015.08.008 PMC4841266

[B209] Yang J. Kim C. S. Tu T. H. Kim M. S. Goto T. Kawada T. (2017). Quercetin protects obesity-induced hypothalamic inflammation by reducing microglia-mediated inflammatory responses via HO-1 induction. *Nutrients* 9:650. 10.3390/nu9070650 28644409 PMC5537770

[B210] Yau P. L. Castro M. G. Tagani A. Tsui W. H. Convit A. (2012). Obesity and metabolic syndrome and functional and structural brain impairments in adolescence. *Pediatrics* 130:E856. 10.1542/peds.2012-0324 22945407 PMC3457620

[B211] Yeh C. W. Yeh S. H. Shie F. S. Lai W. S. Liu H. K. Tzeng T. T. (2015). Impaired cognition and cerebral glucose regulation are associated with astrocyte activation in the parenchyma of metabolically stressed APPswe/PS1dE9 mice. *Neurobiol. Aging* 36:2984. 10.1016/j.neurobiolaging.2015.07.022 26264859

[B212] Yi C. X. Walter M. Gao Y. Pitra S. Legutko B. Kalin S. (2017). TNFalpha drives mitochondrial stress in POMC neurons in obesity. *Nat Commun.* 8:15143. 10.1038/ncomms15143ncomms15143 28489068 PMC5436136

[B213] Yin J. Liu X. C. He Q. Zhou L. J. Yuan Z. Q. Zhao S. Q. (2016). Vps35-dependent recycling of Trem2 regulates microglial function. *Traffic* 17:1286. 10.1111/tra.12451 27717139

[B214] Yirmiya R. Winocur G. Goshen I. (2002). Brain interleukin-1 is involved in spatial memory and passive avoidance conditioning. *Neurobiol. Learn. Mem.* 78:379. 10.1006/nlme.2002.4072 12431424

[B215] Yoo S. Blackshaw S. (2018). Regulation and function of neurogenesis in the adult mammalian hypothalamus. *Prog. Neurobiol.* 170:53. 10.1016/j.pneurobio.2018.04.001 29631023 PMC6173995

[B216] Young A. M. H. Kumasaka N. Calvert F. Hammond T. R. Knights A. Panousis N. (2021). A map of transcriptional heterogeneity and regulatory variation in human microglia. *Nat Genet.* 53:861. 10.1038/s41588-021-00875-210.1038/s41588-021-00875-2 34083789 PMC7610960

[B217] Zabel M. K. Kirsch W. M. (2013). From development to dysfunction: Microglia and the complement cascade in CNS homeostasis. *Ageing Res. Rev.* 12:749. 10.1016/j.arr.2013.02.001 23419464 PMC3700678

[B218] Zenaro E. Pietronigro E. Della Bianca V. Piacentino G. Marongiu L. Budui S. (2015). Neutrophils promote Alzheimer’s disease-like pathology and cognitive decline via LFA-1 integrin. *Nat. Med.* 21:880. 10.1038/nm.3913 26214837

[B219] Zhan Y. Paolicelli R. C. Sforazzini F. Weinhard L. Bolasco G. Pagani F. (2014). Deficient neuron-microglia signaling results in impaired functional brain connectivity and social behavior. *Nat. Neurosci.* 17:400. 10.1038/nn.3641 24487234

[B220] Zhang J. F. Malik A. Choi H. B. Ko R. W. Y. Dissing-Olesen L. MacVicar B. A. (2014). Microglial CR3 activation triggers long-term synaptic depression in the hippocampus via NADPH oxidase. *Neuron* 82:195. 10.1016/j.neuron.2014.01.043 24631344

[B221] Zhang X. Zhang G. Zhang H. Karin M. Bai H. Cai D. (2008). Hypothalamic IKKbeta/NF-kappaB and ER stress link overnutrition to energy imbalance and obesity. *Cell* 135:61. 10.1016/j.cell.2008.07.043 18854155 PMC2586330

